# Mitochondrial dysfunction in Alzheimer’s disease: connecting pathophysiology with neuroimaging

**DOI:** 10.3389/fnagi.2026.1748227

**Published:** 2026-05-29

**Authors:** Jianqin Hu, Jingxue Lai, Bing Zhang, Xuanfei Jiang, Honggang Ma, Ying Liu

**Affiliations:** 1 Huzhou Central Hospital, Affiliated Central Hospital of Huzhou University, Huzhou, Zhejiang, China; 2 School of Medicine & Nursing, Huzhou University, Huzhou, Zhejiang, China

**Keywords:** Alzheimer’s disease, bioenergetics, imaging, mitochondria, mitochondrial dysfunction, neuroimaging

## Abstract

Alzheimer’s disease (AD), primarily characterized by progressive cognitive decline, poses a significant global public health challenge. Emerging evidence indicates that mitochondrial dysfunction plays a central role in AD-related neurodegeneration. This dysfunction manifests as impaired energy metabolism and elevated oxidative stress, and it interacts with β-amyloid (Aβ) and phosphorylated tau (p-tau) pathologies, collectively forming a self-reinforcing vicious cycle. This review systematically explores the mechanisms underlying mitochondrial dysfunction in AD and highlights recent advancements in neuroimaging technologies, such as positron emission tomography (PET), magnetic resonance spectroscopy (MRS), and susceptibility-weighted imaging (SWI), for detecting mitochondrial abnormalities and metabolic disturbances. These multimodal imaging modalities enable the *in vivo* assessment of mitochondrial metabolism, oxidative stress levels, iron deposition, and the integrity of neural networks. Such capabilities not only enhance our understanding of the spatiotemporal progression of mitochondrial pathology in AD but also offer novel tools for early diagnosis, precise patient stratification, and objective evaluation of therapeutic efficacy. Accordingly, this review evaluates the substantial potential of integrating mitochondrial mechanism research with neuroimaging technologies in both foundational research and clinical practice related to AD.

## 1 Introduction

Alzheimer’s disease (AD) is a progressive neurodegenerative disorder characterized by cognitive decline, accounting for approximately 60–80% of dementia cases globally and representing a major public health challenge in aging populations (Alzheimer’s Disease International, 2023). According to the 2023 World Alzheimer Report by Alzheimer’s Disease International (ADI), approximately 55 million individuals currently live with dementia worldwide, a number expected to rise to 139 million by 2050, with the burden increasing most rapidly in low-income countries (Gbd 2019 Stroke Collaborators, 2021). Beyond its substantial economic toll—exceeding USD 1.3 trillion annually—AD imposes prolonged psychological and social stress on family-based caregiving systems (Alzheimer’s Disease International, 2023; Sunderland et al., 2025).

The core pathological features of AD include β-amyloid (Aβ) plaque accumulation, phosphorylated tau (p-tau) hyperphosphorylation leading to neurofibrillary tangles (NFTs), and synaptic loss (Sunderland et al., 2025; Zheng and Wang, 2025). However, therapeutic approaches based on the amyloid hypothesis have encountered significant limitations. Although monoclonal antibodies such as aducanumab and lecanemab effectively reduce cerebral Aβ levels, phase III clinical trials indicate they slow cognitive decline only modestly and carry the risk of amyloid-related imaging abnormalities (ARIA), such as ARIA-E (edema) (van Dyck et al., 2023). Conventional treatments, including cholinesterase inhibitors (e.g., Donepezil) and N-methyl-D-aspartic acid receptor (NMDAR) antagonists (e.g., Memantine), provide temporary symptomatic relief but do not halt disease progression (Birks and Harvey, 2018; McShane et al., 2019).

Emerging research suggests that besides the canonical Aβ and tau axis, other pathological processes (e.g., energy metabolism disorders and oxidative stress induced by mitochondrial dysfunction) may synergistically drive neuronal degeneration via self-reinforcing feedback loops (Munir and du Toit, 2024; Sun J. et al., 2025; Zheng and Wang, 2025). For instance, Aβ oligomers can induce mitochondrial membrane potential (*ΔΨ_*m*_*) collapse, leading to decreased adenosine triphosphate (ATP) production and the simultaneous activation of nicotinamide adenine dinucleotide phosphate (NADPH) oxidase (NOX), which can trigger reactive oxygen species (ROS) bursts that further promote tau hyperphosphorylation and synaptic loss (Ashleigh et al., 2023; McCarty et al., 2021; Norambuena et al., 2018). This complex network of multitarget interactions undermines the efficacy of single-pathway interventions. Importantly, mitochondrial dysfunction is considered central to these pathological mechanisms (Sharma et al., 2021). Among them, mitochondria, as the main source of cellular ROS, their dysfunction can directly lead to aggravated oxidative stress. Oxidative stress, defined by excessive accumulation of ROS and reactive nitrogen species (RNS), is evidenced by elevated biomarkers such as 8-oxo-2′-deoxyguanosine (8-OHdG) and 4-hydroxynonenal (4-HNE) in the cerebrospinal fluid and hippocampal tissue of AD patients (Butterfield and Halliwell, 2019). Notably, oxidative stress can be detected before Aβ plaque formation, suggesting that it may serve as an early pathogenic trigger rather than a downstream consequence (Johnson et al., 2020). The resulting cellular damage involves mitochondrial DNA (mtDNA) oxidation and fragmentation, lipid peroxidation–induced membrane rigidity, and proteasomal overload caused by protein misfolding (Picca et al., 2020). Moreover, mitochondria, the primary intracellular source of ROS, engage in a vicious cycle with oxidative stress: Aβ has been shown to directly target mitochondrial complex IV, impairing electron transport chain (ETC) function and amplifying ROS production (Du et al., 2010). Elevated ROS levels further induce mtDNA mutations and disrupt mitochondrial autophagy (mitophagy), potentially culminating in cellular energy crises and neuronal apoptosis (Schofield and Schafer, 2021; Swerdlow et al., 2010). This “mitochondria–oxidative stress–Aβ and tau” axis may present promising new therapeutic targets for AD intervention. However, the dynamic origin of this complex network—specifically, whether oxidative stress or Aβ pathology precedes the other—remains a critical issue in the field. On the one hand, studies suggest that oxidative stress can be detected prior to the formation of Aβ plaques, implying it may serve as an early trigger rather than a mere downstream consequence (Butterfield and Halliwell, 2019). This perspective has been supported by imaging findings in animal models, such as the observation of mitochondrial respiratory dysfunction even at the stage when only soluble Aβ is present (Norambuena et al., 2024). On the other hand, evidence indicates that Aβ oligomers themselves can directly damage mitochondria and induce ROS bursts, thereby initiating a self-reinforcing vicious cycle (Mustaly-Kalimi et al., 2025). This apparent contradiction highlights the systemic and nonlinear nature of the AD pathological mechanism: oxidative stress and Aβ deposition may not represent a simple linear causal relationship but rather a co-pathological process that is mutually driving and synergistically amplified at different stages of the disease. Therefore, while elucidating the initial link in this cycle is crucial, disrupting the entire pathological network may prove more effective as a therapeutic strategy. This also underscores the critical importance of identifying and intervening on this axis of dysfunction before symptom onset.

Against this background, strategies that can adopt early noninvasive or minimally invasive methods to detect mitochondrial and oxidative stress abnormalities may be crucial for the early diagnosis and early intervention of AD. Among these, neuroimaging techniques have emerged as key tools for analyzing the above pathological dynamics. By integrating multimodal imaging [such as positron emission tomography (PET), magnetic resonance imaging (MRI), and magnetic resonance spectroscopy (MRS)], researchers can noninvasively evaluate intracranial oxidative stress levels, mitochondrial metabolic flux, and neural network remodeling processes, thereby revealing the heterogeneous characteristics of AD subtypes and guiding precision therapy (Berkowitz, 2018). For example, fludeoxyglucose-18 (^18^F-FDG)-PET has shown that reduced glucose metabolism in the temporal and parietal lobes of patients with AD may be related to decreased mitochondrial complex activity, while functional magnetic resonance imaging (fMRI) has shown that the connectivity strength of the default mode network (DMN) is positively correlated with mitochondrial membrane potential (Geary, 2021; Terada et al., 2020). These findings not only deepen the understanding of the pathological mechanisms of AD but also provide a scientific foundation for the development of novel therapies targeting energy metabolism and redox balance (Li et al., 2024; Neto et al., 2024).

This review systematically examines the mechanistic roles of mitochondrial dysfunction in AD and synthesizes recent advances in imaging-based diagnostics of mitochondrial abnormalities and metabolic impairment. By integrating molecular insights with imaging evidence, it evaluates the potential for clinical translation and outlines prospective directions for future research and application. For ease of comparison, Table 1 summarizes the main neuroimaging methods discussed in the text, along with their detection targets, advantages, disadvantages, and other aspects.

**TABLE 1:** Imaging-related techniques.

Technical name	Detection target	Suitable for early AD detection?	Advantages	Disadvantages/limitations	References
^31^P-MRS	High-energy phosphate metabolites (e.g., PCr, ATP, Pi); Cerebral energy metabolism.	Yes (Research tool).	The only noninvasive technique for *in vivo* detection of OXPHOS metabolites.	Low sensitivity, prolonged scanning time, poor spatial resolution, lack of standardization.	(Jett et al., 2023; Qiao et al., 2006; Rietzler et al., 2022)
fMRI	Changes in CBF and blood oxygenation; Indirect assessment of brain metabolism and neural network activity.	Potential (Research stage).	Noninvasive, balanced spatiotemporal resolution, whole-brain imaging.	Complex signals susceptible to noise; Lack of standardized individual-specific analysis; Limited routine clinical applications.	(Butterfield and Halliwell, 2019; Vachha et al., 2025; Zhao Y. et al., 2025)
^31^P-MT-MRSI	ATP metabolic kinetic parameters.	No (Primarily a preclinical research tool).	Provides a dynamic perspective on cellular energy homeostasis.	Technically complex, limited clinical application.	(Prasuhn et al., 2022; Tachrount et al., 2025)
^18^F-BCPP-EF PET	Mitochondrial ETC complex I activity; Cerebral energy metabolism status.	Potential (Early clinical research stage).	A novel tool for *in vivo* assessment; Correlates with early tau deposition in AD.	Heterogeneous performance across disease models; Early stage of clinical translation.	(Tsukada et al., 2014; Yamagishi et al., 2021)
bNIRS	CCO oxidation state; OXPHOS activity.	Not explicitly stated.	Utilizes spectral differences to separate CCO signals.	Requires physiological challenges to induce signal changes; Diverse data processing algorithms, insufficient standardization.	(Prasuhn et al., 2022)
^13^C-MRI	Metabolite ratios (e.g., lactate/pyruvate); Indirectly reflects redox state/ROS levels.	Potential (Under clinical translation).	Can indirectly assess oxidative stress status.	High equipment requirements, complex tracer preparation; Mainstream techniques are costly and time-consuming.	(He et al., 2024; Sharma et al., 2024; Wodtke et al., 2025)
^11^C-acetate PET	Cellular oxidative metabolism level.	Not explicitly stated (Mainly applied in tumor imaging, limited use in the nervous system).	Can reflect cellular oxidative metabolism.	Relatively limited application in the nervous system, especially for directly evaluating mitochondrial function; Few related reports.	(Song et al., 2025)
FDG-PET	CMRglc.	Yes (Used for early clinical assessment).	Can detect early reductions in brain glucose metabolism.	Cannot evaluate the OXPHOS process; High cost and radiation exposure.	(de Leon et al., 1983; Foster et al., 1983; Friedland et al., 1983; Haidar et al., 2023; Vaishnavi et al., 2010)
^1^H-MRSI	Low-molecular-weight antioxidants (e.g., GSH).	Yes (Validated in studies on early AD/MCI stages).	Can quantitatively detect key antioxidant GSH and evaluate supplementation therapy effects.	Measurement reproducibility affected by spectral peak overlap and technical variations.	(Detcheverry et al., 2025; Mandal et al., 2015; Mandal et al., 2019; Mischley et al., 2016)
^62^Cu-ATSM PET	Cellular hyper-reduced state; Indirect imaging of oxidative stress-related pathology.	Potential (Research stage).	Enables *in vivo* visualization of oxidative stress-related pathology.	Extremely short half-life of ^62^Cu limits clinical accessibility; Small sample sizes in existing studies.	(Donnelly et al., 2012; Holland et al., 2009; Ikawa et al., 2020; Yoshii et al., 2012)
SWI	Local magnetic field inhomogeneity caused by nonheme iron deposition; Indirectly reflects oxidative stress.	Not explicitly stated.	Can detect iron deposition associated with oxidative stress.	Traditional SWI cannot distinguish between Fe^2+^ (involved in Fenton reaction) and the relatively inert Fe^3 +^.	(Biasiotto et al., 2019; Dietrich et al., 2017)
QUEST-MRI	Changes in relaxation rate after antioxidant treatment; Reflects the “overall oxidative state” of tissue.	Potential (Preclinical tool with translational potential).	No need for exogenous contrast agent; High spatial resolution; Compatible with standard MRI hardware.	Cannot identify specific ROS types; Not yet systematically applied in AD patients.	(Berkowitz, 2018; Hollen et al., 2022; Prasuhn et al., 2022)
^18^F-FEDV PET	Central nervous system oxidative stress (broad-spectrum RONS reactivity).	Potential (Preclinical research stage).	Can be used for direct imaging of oxidative stress *in vivo*.	Low automated synthesis yield; Lacks unified imaging and quantification standards.	(Ghosh et al., 2025; Wilde et al., 2025)
^18^F-ROStrace	The level of oxidative stress reflected by ROS, particularly superoxide anions, in the brain.	Potential (Preclinical tool with translational potential).	The first PET tracer for direct quantitative and dynamic visualization of ROS in the brain; exhibits high selectivity for superoxide anions and low background uptake in normal tissues; shows a high degree of consistency with signal levels of oxidative stress-related biological pathological indicators.	Unable to distinguish between mitochondrial and cytoplasmic ROS sources; easily influenced by blood perfusion and systemic inflammatory status.	(Gallagher et al., 2025; Park et al., 2026)
QSM	Regional brain iron concentration (e.g., hippocampus).	Yes (Applied in research).	No radiation risk, suitable for repeat examinations.	High requirements for magnetic field homogeneity; Technique still under optimization.	(Onukwufor et al., 2022; Stone and Blockley, 2017)
NIRF	ROS molecules.	Potential (Preclinical stage, e.g., with CRANAD-61 probe).	Enables specific imaging of ROS.	Limited tissue penetration depth, a bottleneck for clinical translation.	(Yang et al., 2017)
CRANAD-61	Specific imaging of ROS; Distinguishes “active” from “resting” plaques in AD.	Potential (Preclinical stage).	Allows micro- and macro-level imaging with higher reliability.	Relies on NIRF technology, similarly limited by penetration depth.	(Yang et al., 2019; Yang et al., 2017)
^2^P-FLIM	Mitochondrial respiratory response; NADH metabolism.	Yes (Animal models confirm it precedes plaque deposition).	Provides dynamic imaging evidence in AD animal models, confirming mitochondrial dysfunction precedes Aβ deposition.	Applied in preclinical animal models; human application not mentioned.	(Norambuena et al., 2024)
MP-PAM	Vascular oxygen consumption defects.	Yes (Animal models confirm it precedes plaque deposition).	Synchronized with ^2^P-FLIM, confirming mitochondrial dysfunction precedes Aβ deposition.	Applied in preclinical animal models; human application not mentioned.	(Norambuena et al., 2024)
DTI	White matter microstructure damage (via FA/MD parameters); Reflects tau-mediated mitochondrial transport impairment.	Yes (Can detect white matter network damage related to cognitive impairment).	Can detect white matter ultrastructural damage.	Specific disadvantages not explicitly mentioned.	(Griffiths and Grant, 2023; Jett et al., 2023)
rs-fMRI	Brain network connectivity (e.g., default mode network abnormalities).	Yes (Can detect early neural network abnormalities related to Aβ and tau pathology).	Shows network disruption consistent with Aβ and tau-induced mitochondrial dysfunction.	Complex signal interpretation; Limited routine clinical applications.	(Jett et al., 2023; Warren and Moustafa, 2023)
^18^F-AV45 PET	Aβ plaque deposition.	Yes (Clinically used for early diagnosis and differential diagnosis).	Has established clear clinical guidelines; a critical step in screening for disease-modifying therapies.	Nonspecific binding to white matter; Low sensitivity for detecting low amyloid loads; Long scan times.	(Clark et al., 2011; Rabinovici et al., 2025; Sabri et al., 2015)
High-Resolution EM (3DEM, SBEM, FIB-SEM)	Subcellular ultrastructural abnormalities (e.g., mitochondrial cristae membrane rupture, fragmentation, autophagosomes).	No (Currently confined to preclinical research).	Gold standard for revealing ultrastructural abnormalities; Key for evaluating potential therapeutic strategies.	Extremely complex and time-consuming sample preparation and data analysis workflows; Lack of standardized protocols.	(Choi et al., 2020; Colasuonno et al., 2017; Ju et al., 2023; Panes et al., 2023)
QSM+qBOLD Model	OEF, CBF, tissue oxygen metabolism.	Not explicitly stated.	Enables more comprehensive assessment of brain tissue oxygen metabolism and circulation.	Under development.	(Biondetti et al., 2023)

^31^P-MRS, phosphorus-31 magnetic resonance spectroscopy; fMRI, functional magnetic resonance imaging;

^31^P-MT-MRSI, phosphorus-31 magnetization transfer magnetic resonance spectroscopic imaging;

^18^F-BCPP-EF,

^18^F-2-tert-butyl-4-chloro-5-{6-[2-(2-fluoroethoxy)-ethoxy]-pyridin-3-ylmethoxy-2H-pyridazin-3-one;

^13^C-MRI, hyperpolarized carbon-13 magnetic resonance imaging;

^11^C-acetate PET, carbon-11 acetate positron emission tomography imaging; FDG-PET, fluorodeoxyglucose positron emission tomography;

^1^H-MRSI, proton magnetic resonance spectroscopy imaging;

^62^Cu-ATSM, copper-62 diacetyl-bis(N4-methylthiosemicarbazone); SWI, susceptibility-weighted imaging; QUEST-MRI, QUEnching-assiSTed magnetic resonance imaging;

^18^F-FEDV, 2-deoxy-2-[^18^F]fluoro-α-D-arabinofuranosylvinyluracil; QSM, quantitative susceptibility mapping; NIRF, near-infrared fluorescence imaging;

^2^P-FLIM, two-photon fluorescence lifetime imaging; MP-PAM, multi-parameter photoacoustic microscopy imaging; DTI, diffusion tensor imaging; rs-fMRI, resting-state functional magnetic resonance imaging;

^18^F-AV45 PET, [^18^F]florbetaben positron emission tomography; 3DEM, three-dimensional electron microscopy; SBEM, serial block-face scanning electron microscopy; FIB-SEM, focused ion beam scanning electron microscope; PCr, phosphocreatine; ATP, adenosine triphosphate; Pi, inorganic phosphate; OXPHOS, oxidative phosphorylation; CCO, cytochrome c oxidase; GSH, glutathione; ROS, reactive oxygen species; RONS, reactive oxygen and nitrogen species; BBB, blood-brain barrier; Aβ, β-amyloid; AD, Alzheimer’s disease; CMRO2, cortical microvascular oxygenation; FA/MD, fractional anisotropy/mean diffusivity; OEF, oxygen extraction fraction; EM, electron microscopy; qBOLD, quantitative blood oxygenation level dependent; CRANAD-61, 2-(4′-dimethylaminophenyl)-benzothiazolyl-derived cyanine dye; ETC, electron transport chain; CBF, cerebral blood flow; PET, positron emission tomography; CMRglc, cerebral metabolic rate of glucose; MCI, mild cognitive impairment.

## 2 Physiological functions of mitochondria and markers of signal transduction

Mitochondria are highly dynamic, double-membrane organelles found in eukaryotic cells. They have long been recognized as the “cell powerhouses” due to their essential role in synthesizing ATP efficiently through the tricarboxylic acid (TCA) cycle and the oxidative phosphorylation (OXPHOS) system (Yu and Pekkurnaz, 2018). Extensive studies have validated this classical view: mitochondria integrate the end products of carbohydrate, lipid and protein metabolism—via pathways such as β-oxidation and amino acid catabolism—and generate over 90% of cellular ATP via the ETC, establishing them as central hubs in cellular energy metabolism (Alan and Scorrano, 2022).

However, advancing research has revealed that mitochondria function as more than mere energy producers; they are increasingly regarded as cellular “processors.” Alongside the nucleus and other organelles, they comprise the mitochondrial information processing system (MIPS), which plays a critical role in determining cell fate, regulating metabolism, and maintaining systemic homeostasis (Picard and Shirihai, 2022). MIPS is believed to operate through three tiers of information flow involving a wide array of biologically significant markers. Mitochondria initially sense signals from small molecules (e.g., gases and ions) and biological macromolecules (e.g., metabolites, proteins, and lipids), including mtDNA, as well as physical parameters, such as temperature and mitochondrial membrane potential. These inputs are then integrated into complex signaling networks through mechanisms including ligand-receptor binding and second messenger cascades. Subsequently, mitochondria transmit output signals, such as energy carriers, metabolites, ROS, mtDNA, and RNA, thermal cues, physical interactions, peptides, steroids, calcium ions, and vesicles, to coordinate diverse cellular processes (Picard and Shirihai, 2022). These include apoptosis, calcium homeostasis, the mitochondrial unfolded protein response (mtUPR), mtDNA signaling, ROS signaling, and broader metabolic pathways. Mitochondrial signaling also influences epigenetic regulation, facilitating communication at multiple levels: molecular, subcellular (inter-organelle), cellular (intercellular), and endocrine (organ/system-level) (Picard and Shirihai, 2022). Supplementary Table S1 summarizes the key signaling pathways and principal biomarkers associated with mitochondrial function.

Importantly, mitochondrial function is heterogeneous across tissues and cell types. In the brain, neurons exhibit a particularly high dependence on mitochondria, which are densely localized at the synaptic sites. These mitochondria are essential for maintaining neuronal membrane excitability, synaptic transmission, and plasticity (Monzel et al., 2023; Pekkurnaz and Wang, 2022), thereby supporting sustained neuronal activity, synaptic adaptability, and cell survival.

## 3 Core mechanisms of mitochondrial dysfunction and imaging evidence in AD

As previously mentioned, mitochondrial dysfunction in AD has garnered growing attention. The core upstream mechanism is primarily defined by mitochondrial bioenergetic deficits, whereas downstream consequences encompass mitochondrial interactions with Aβ and p-tau, impaired mitochondrial biogenesis, disrupted calcium homeostasis, and other aberrant molecular pathways. In recent years, advances in neuroimaging techniques, such as PET, MRS, and susceptibility-weighted imaging (SWI), in conjunction with key biomarkers of mitochondrial dysfunction, such as cerebral ATP and its metabolite phosphocreatine (PCr), iron accumulation, and cerebral glucose metabolic rate and other parameters, have enabled multi-level, noninvasive assessments. These approaches facilitate a deeper understanding of the mechanisms underlying mitochondrial dysfunction in AD and their association with clinical manifestations (Jett et al., 2023; Onukwufor et al., 2022; Swerdlow, 2018). Furthermore, neuroimaging has provided compelling support for the “mitochondrial cascade hypothesis.” By linking microscopic pathological processes to macroscopic imaging phenotypes, these imaging modalities serve as robust tools for translational research and hold promise as potential surrogate endpoints for early diagnosis, monitoring disease progression, and evaluating therapeutic interventions for AD.

### 3.1 Mitochondrial bioenergetic defects in AD

**FIGURE 1:**
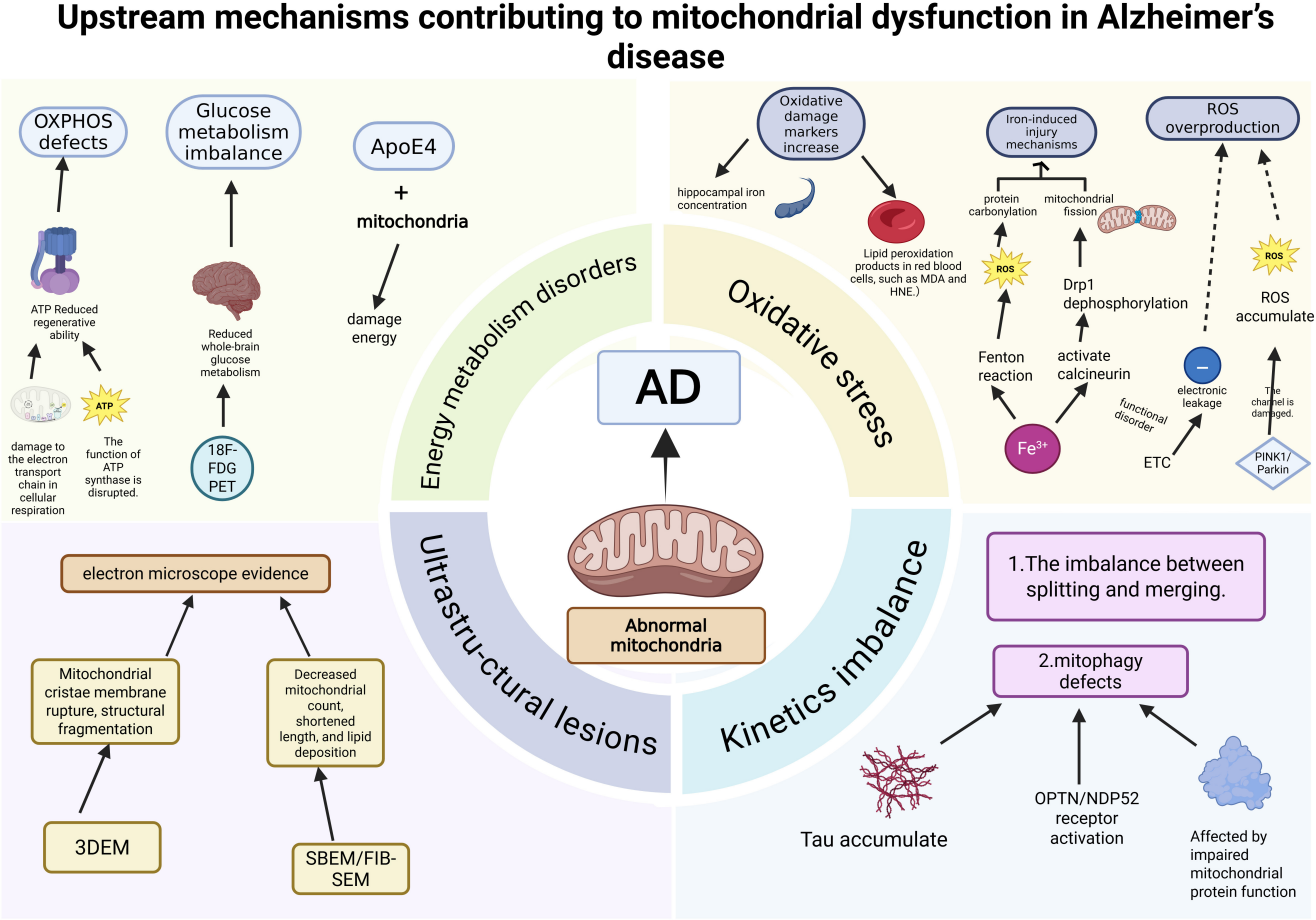
Upstream mechanisms contributing to mitochondrial dysfunction in Alzheimer’s disease. In Alzheimer’s disease (AD), mitochondrial abnormalities involve multidimensional mechanisms, where upstream mechanisms specifically manifest as: in terms of energy metabolism, oxidative phosphorylation (OXPHOS) is significantly impaired, characterized by a decline in adenosine triphosphate (ATP) regeneration capacity and a widespread reduction in the activity of electron transport chain (ETC) complexes. Additionally, ^18^F-FDG PET confirms a decrease in cerebral metabolic rate of glucose (CMRglc), but this result cannot be directly equated to OXPHOS dysfunction. Moreover, apolipoprotein E4 (ApoE4) impairs energy production by reducing membrane potential and causing mitochondrial fragmentation. Enhanced oxidative stress is a critical factor, with increased iron concentration in the hippocampus of AD patients, which, through the Fenton reaction, produces reactive oxygen species (ROS), inducing lipid peroxidation (e.g., increased levels of MDA, HNE, etc.) and protein carbonylation. Simultaneously, Fe^3 +^ activates calmodulin-dependent phosphatase (CDPP) leading to dephosphorylation of dynamin-related protein 1 (Drp1), resulting in excessive mitochondrial fragmentation. Furthermore, ETC dysfunction causes electron leakage and PINK1/Parkin pathway impairment, further exacerbating the overproduction of ROS. Additionally, electron microscopy (EM) reveals ultrastructural lesions in mitochondria of AD patients, with three-dimensional electron microscopy (3DEM) showing cristae disruption and fragmentation, and serial block-face electron microscopy (SBEM) confirming a reduction in number, shortening in length, and lipid deposition. Kinetically, there is an imbalance in fission-fusion, as well as tau accumulation, activation of OPTN/NDP52 receptors, and impaired mitochondrial protein function leading to defective mitophagy. (created with BioRender.com).

In AD, mitochondrial energy production decreases significantly, leading to dysregulation of intracellular energy metabolism. This bioenergetic defect not only affects the normal function of neurons but may also exacerbate the pathological accumulation of Aβ and p-tau. These bioenergetic disorders in AD mainly include energy metabolism disorders, oxidative stress, ultrastructural lesions, and dynamic imbalance (Li et al., 2022). Figure 1 describes the specific process of this upstream mechanism.

#### 3.1.1 Energy metabolism disorders

As the body’s most energy-intensive organ, the brain relies heavily on mitochondria to produce ATP through OXPHOS. However, AD patients often experience insufficient energy supply in the brain, and this metabolic deficit can directly cause neuronal dysfunction and cognitive decline. The physiological process of OXPHOS involves mitochondria in eukaryotic cells using electrons generated by aerobic respiration to phosphorylate adenosine diphosphate (ADP) into ATP (Pan et al., 2022). In this process, the ATP generated from the combination of ADP and inorganic phosphate (Pi) is not isolated but further participates in critical physiological pathways, such as PCr, serving as the core material basis for energy transfer and utilization. Among them, PCr acts as an “immediate energy reservoir” for ATP, rapidly regenerating it to maintain a stable brain energy supply, while Pi serves as a marker of energy consumption. Therefore, the PCr/ATP ratio reflects energy reserves and regeneration efficiency, whereas the PCr/Pi ratio indicates the balance between cellular energy supply and demand. Both ratios are key indicators for assessing the health of cerebral cellular energy metabolism (Chen et al., 2019; Gordji-Nejad et al., 2018; Yuksel et al., 2015).

To accurately evaluate the aforementioned indicators, phosphorus-31 magnetic resonance spectroscopy (^31^P-MRS), as the only noninvasive technique for *in vivo* detection of OXPHOS metabolites, has been widely applied in clinical trials and is currently an indispensable research tool for *in vivo* studies of cerebral energy metabolism. However, its clinical application is primarily confined to the scientific research field and has not yet become a routine diagnostic procedure (Jett et al., 2023). By quantifying metabolites such as PCr, ATP, and Pi, ^31^P-MRS can provide key energy metabolism indicators and serve as one of the important imaging methods for detecting energy metabolism disorders in the brain of AD patients (Parasoglou et al., 2022; Zhao S. et al., 2025). Notably, this technique uses magnetic transfer to noninvasively and accurately measure biochemical reaction rates and enzyme activity by detecting changes in the magnetization of reactants (e.g., Pi) (Ren et al., 2016; Ren et al., 2017). However, this technique is limited by low sensitivity, a high detection limit, significant spectral overlap, and low spatial resolution. These factors contribute to a low signal-to-noise ratio (SNR), prolonged scanning times, and difficulty in accurately quantifying small regions like the hippocampus. Additionally, insufficient standardization of protocols across studies further restricts the broad comparability of results (Jett et al., 2023; Rietzler et al., 2022). In addition, other neuroimaging techniques can be employed to assess OXPHOS-related brain metabolic levels. For instance, phosphorus-31 magnetization transfer magnetic resonance spectroscopic imaging (^31^P-MT-MRSI) combined with magnetization transfer pulses can quantify ATP metabolic kinetic parameters (such as Pi↔ATP, Pi↔PCr, and PCr↔ATP), an approach suitable for evaluating the regulatory effects of drugs on energy metabolism. This technique provides a dynamic perspective on cellular energy homeostasis; however, due to its high complexity, its clinical application is limited, and it is currently primarily used as a preclinical research tool (Tachrount et al., 2025). Future technological advancements should focus on utilizing ultra-high field strength (e.g., 7T) MRI to enhance SNR and resolution, developing rapid imaging sequences, and establishing standardized analytical workflows. Additionally, prospective studies should clarify the biomarker value of these techniques in the early diagnosis and efficacy evaluation of AD (Jett et al., 2023; Rietzler et al., 2022).

In the progression of AD, abnormal brain glucose metabolism is considered one of the core pathological features in early stages. As early as the 1980s, researchers used fluorodeoxyglucose positron emission tomography (FDG-PET) to detect neuroimaging biomarkers—specifically, reduced glucose metabolism rates in designated brain regions, and found that AD patients had lower glucose utilization in the brain compared to healthy controls. This finding not only generated widespread interest in brain glucose metabolism but also accelerated the clinical exploration of FDG-PET applications (de Leon et al., 1983; Foster et al., 1983; Friedland et al., 1983). Currently, FDG-PET has been applied in early dementia assessment and differential diagnosis. It can be used for direct measurement of glucose metabolism processes, which are highly sensitive to changes in neuronal and synaptic activity, thereby reflecting degenerative or pathological alterations. However, despite its advantages, FDG-PET remains prohibitively expensive and involves high radiation exposure (Haidar et al., 2023). Moreover, while FDG-PET can detect early reductions in cerebral metabolic rate of glucose (CMRglc), it cannot evaluate OXPHOS processes—which account for 90% of brain ATP production (Chia et al., 2025; Vaishnavi et al., 2010). Therefore, combining FDG-PET with ^31^P-MRS is essential for a comprehensive understanding of AD’s energy imbalance mechanisms.

Recently, researchers have developed a novel approach to indirectly assess brain metabolism through fMRI (Zhao Y. et al., 2025). Traditional MRI can provide high-resolution, detailed images of brain structure; fMRI, on the other hand, maps brain activity by detecting changes in blood flow and blood oxygen levels, which are closely related to neural activity. This technology has become a key tool for fundamental brain science research and the exploration of cognitive neurodisease mechanisms due to its advantages: it is noninvasive, offers balanced spatiotemporal resolution, has high reproducibility, and enables whole-brain imaging (Xia and He, 2022). Furthermore, this technology can reveal differences in metabolic activity and neurotransmitter levels between different brain regions, identify metabolic alterations in brain tumors, and successfully localize and characterize lesions in patients with multiple sclerosis (MS). Patients only need to remain in the fMRI scanner for several minutes to complete the examination (Zhao Y. et al., 2025). This breakthrough fMRI technology maps brain metabolism with unparalleled precision, playing a significant role in clinical metabolic imaging. However, the widespread clinical application of fMRI in routine diagnosis and treatment still faces challenges (Vachha et al., 2025). Currently, its clinical applications are largely confined to a few scenarios, such as preoperative planning in neurosurgery (e.g., functional zone localization and epileptogenic focus detection) (Vachha et al., 2025). Key factors hindering its widespread clinical application include: complex fMRI signals susceptible to noise interference (Chan et al., 2024); lack of standardized and reliable individual-specific analytical techniques and interpretation methods (Reeves et al., 2025); and the lack of efficient automated analysis software for complex data processing workflows in clinical settings (Ma et al., 2026). Looking ahead, key directions for advancing the clinical translation of fMRI include: developing higher-field-strength magnetic resonance systems (e.g., 5T, 7T, or even 10.5T) to enhance SNR and spatial resolution (Qu et al., 2026); creating more advanced sequences and artificial intelligence (AI) algorithms to reduce scanning time and improve quantification accuracy (Khajehim et al., 2021); and supporting broader applications through the establishment of large-scale brain imaging databases and standardized workflows (Lee et al., 2022).

Furthermore, PET tracers targeting mitochondrial ETC complex I, such as ^18^F-2-tert-butyl-4-chloro-5-{6-[2-(2-fluoroethoxy)-ethoxy]-pyridin-3-ylmethoxy-2H-pyridazin-3-one (^18^F-BCPP-EF) (Tsukada et al., 2014). This technology has demonstrated potential to correlating with p-tau deposition in AD and exhibits good reproducibility, thus attracting significant attention in clinical research on AD (Baier et al., 2025; Mattsson-Carlgren et al., 2020). However, the standardization level of its clinical application and cross-center consistency still require further validation, posing challenges for its promotion to clinical practice. A study indicated that while a trend of reduced ^18^F-BCPP-EF binding levels was observed in multiple key brain regions in Parkinson’s disease (PD) patients, this trend was not statistically significant, and longitudinal assessments only revealed trend changes. The differences observed in AD and PD studies suggest that the pattern of mitochondrial complex I dysfunction may be disease-specific, or the sensitivity of this technology may be influenced by the pathological background of different diseases (Terada et al., 2021; Tsukada et al., 2014). Another technique is broadband near-infrared spectroscopy (bNIRS), which detects OXPHOS activity by utilizing the absorption spectral differences of cytochrome c oxidase (CCO) at different oxidation states. It can separate CCO/oxidized cytochrome c oxidase (oxCCO) signals from hemoglobin signals. However, signal changes need to be induced through physiological challenges (such as variations in respiratory rate) rather than direct detection in the resting state (Lange et al., 2019). Furthermore, the current data processing algorithms are diverse and lack standardization, which further limits their feasibility for clinical application (Prasuhn et al., 2022). Nevertheless, the imaging findings obtained based on this technology remain of significant importance, as they not only provide imaging evidence for the aforementioned molecular mechanisms (such as OXPHOS dysfunction, abnormal PCr/ATP ratio) but also highlight the indispensable role of multimodal imaging in revealing the energy crisis in AD.

This section provides a detailed discussion on the manifestations of mitochondrial bioenergetic defects in AD, specifically focusing on energy metabolism disorders (Li et al., 2022). Corresponding imaging techniques offer critical *in vivo* evidence for these mechanisms. Regarding the current state and the advantages and disadvantages of these technologies, ^31^P-MRS remains an indispensable *in vivo* tool for studying cerebral energy metabolism, enabling quantitative measurement of metabolites such as PCr and ATP. However, its clinical application is primarily confined to research due to low sensitivity, prolonged scanning time, poor spatial resolution, and lack of standardization (Jett et al., 2023; Qiao et al., 2006). ^31^P-MT-MRSI can provide ATP metabolic kinetics but is technically complex and similarly serves mainly as a preclinical research tool (Tachrount et al., 2025). FDG-PET can detect early reductions in CMRglc and has been utilized for early clinical assessment. However, it cannot directly evaluate the ATP-generating OXPHOS process in the brain, and it is associated with high costs and radiation exposure (Haidar et al., 2023; Vaishnavi et al., 2010). fMRI can indirectly assess brain metabolism and neural network activity, but signal interpretation is complex, and its clinical applications are mostly limited to a few scenarios such as preoperative planning (Zhao Y. et al., 2025). PET tracers targeting mitochondrial complex I (e.g., ^18^F-BCPP-EF) provide a novel tool for *in vivo* evaluation of brain energy metabolism, demonstrating potential for disease-specific applications, but they are still in the clinical research stage (Mansur et al., 2020; Shatalina et al., 2025). bNIRS reflects OXPHOS activity by detecting CCO signals, but it requires physiological challenges to induce signal changes and lacks standardized data processing (Lange et al., 2019; Prasuhn et al., 2022). However, there are still some research gaps, such as the difficulty in accurately quantifying energy metabolism changes in small brain regions like the hippocampus with existing technologies, and the lack of longitudinal studies to reveal the spatiotemporal evolution of energy metabolism disorders and Aβ and tau pathology. Future efforts should focus on improving the SNR and resolution of ^31^P-MRS using ultra-high field MRI, developing rapid imaging sequences and standardized analysis workflows, and promoting multimodal fusion (e.g., combining ^31^P-MRS with FDG-PET) to comprehensively elucidate the mechanisms of early energy imbalance in AD and its value as a biomarker for therapeutic efficacy evaluation (Jett et al., 2023; Rietzler et al., 2022).

#### 3.1.2 Oxidative stress and iron metabolism disorders

Mitochondrial dysfunction in AD leads to excessive production of ROS, often accompanied by dysregulation of iron metabolism in the brain. Iron ions catalyze ROS generation through the Fenton reaction, and elevated ROS further impair mitochondrial function, promote p-tau phosphorylation, and form a self-reinforcing pathological cycle (Misrani et al., 2021; Onukwufor et al., 2022; Tönnies and Trushina, 2017). The significant increase in oxidative stress is accompanied by elevated biomarkers of oxidative damage, including lipid peroxidation products [e.g., malondialdehyde (MDA)] in brain tissue and red blood cells, as well as detectable biomarkers such as 8-OHdG and 4-HNE in cerebrospinal fluid and hippocampal tissue of AD patients (Munir and du Toit, 2024; Onukwufor et al., 2022).

Neuroimaging provides several indirect approaches for measuring ROS levels. For instance, quantitative susceptibility mapping (QSM) reveals significantly elevated iron concentrations in the hippocampus of AD patients, suggesting a pathological environment conducive to ROS generation (Onukwufor et al., 2022). Originally developed as a phase-reconstruction MRI method, QSM has since evolved into a fusion model that integrates phase (QSM) and amplitude [quantitative blood oxygenation level-dependent, quantitative blood oxygenation level dependent (qBOLD)] data—referred to as the QSM+qBOLD (QQ) model—to more accurately estimate oxygen extraction fraction (OEF) (Stone and Blockley, 2017). Unlike PET scans requiring radioactive tracers, QSM eliminates radiation risks, making it clinically preferable for repeat examinations. It simultaneously measures OEF, cerebral blood flow (CBF), and cortical microvascular oxygenation (CMRO_2_), providing comprehensive assessment of brain tissue oxygen metabolism and circulation (Biondetti et al., 2023). However, QSM technology has stringent requirements for magnetic field uniformity. Therefore, although the technology can assess oxygen metabolism and blood flow changes in the whole brain and deep gray matter (such as the hippocampus), it remains in the optimization phase. Future improvements will enhance its accuracy through vascular model refinement, optimized individualized hematocrit (Hct) measurement methods, and extended arterial spin labeling (ASL) pulsed-late detection (PLD) duration (Yang et al., 2023). Additionally, superparamagnetic hyperpolarized carbon-13 magnetic resonance imaging (^13^C-MRI) technology has been developed. It uses tracers such as [1-^13^C] pyruvate, and reflects the cytoplasmic reduced NADH/NAD^+^ ratio, i.e., the redox state, through the ratio of its metabolic products (such as lactate/pyruvate ratio). Given the close correlation between redox status and ROS levels, this technique can indirectly evaluate the oxidative stress status. However, this technology still presents several limitations during measurement, such as high equipment requirements and complex tracer preparation (Sharma et al., 2024). It is gratifying to note that, substantial progress has been made in the clinical translation of this technology. Clinical polarizers based on dissolved dynamic nuclear polarization (dDNP) have supported human studies at over 24 research centers worldwide (Wodtke et al., 2025). Although widespread clinical application has not yet been achieved, the imaging protocol using its core tracer [1-^13^C]pyruvate has been recognized by the academic community as having potential applications in neurological disorders such as gliomas and AD (He et al., 2024). However, significant potential challenges remain for its widespread adoption: mainstream dDNP techniques are costly and time-consuming, while alternative hydrogen-based techniques [e.g., parahydrogen induced polarization (PHIP), signal amplification by reversible exchange (SABRE)] are faster and more economical, but require specific optimization for different molecular probes (Hsieh et al., 2025). Key future focuses include: developing more novel probes (e.g., [2-^13^C]pyruvate) to expand the range of metabolically monitorable pathways (Hsieh et al., 2025); conducting spectral studies at higher magnetic field strengths (e.g., 7T) to improve SNRs (He et al., 2024); designing dedicated magnetic resonance sequences for hyperpolarized ^13^C (He et al., 2024); and optimizing data acquisition and analysis workflows using advanced methods such as AI (Hsieh et al., 2025; Wodtke et al., 2025). As an emerging imaging modality, QUEnching-assiSTed magnetic resonance imaging (QUEST-MRI) employs a unique indirect detection strategy. It measures changes in the body’s relaxation rate (R1) following antioxidant treatment to reflect the quantity of paramagnetic ROS molecules (Berkowitz, 2018). Its core advantages are no need for exogenous contrast agent, high spatial resolution and compatibility with standard MRI hardware. These features make QUEST-MRI particularly promising for expanding our understanding of regional specificity in oxidative stress, aiding evaluation of novel antioxidant therapies in animal models or patients, and potentially guiding personalized treatment decisions (Prasuhn et al., 2022). Currently, this technology is primarily utilized as a preclinical research tool. Leveraging its core advantages, it is recognized as possessing significant clinical translation potential (Hollen et al., 2022). However, the method measures overall relaxation rate changes induced by antioxidants, reflecting the “overall oxidative state” of tissues, with limited capacity to identify specific types of ROS (Hollen et al., 2022). To date, systematic application studies in AD patients remain unexplored. Whether it can be employed for dynamic monitoring of oxidative stress changes during AD progression, as well as its association with core pathological markers such as Aβ and tau, remains to be investigated. Future efforts should focus on advancing its translation into clinical trials, establishing standardized protocols, and validating its potential as a biomarker for disease progression or therapeutic efficacy (Bøgh et al., 2024).

Further expanding, iron-sensitive MRI techniques such as SWI indirectly reflect oxidative stress by detecting local magnetic field inhomogeneities caused by nonheme iron. Its principle is mainly related to iron salts catalyzing the Fenton reaction of H_2_O_2_ [which is also a major source of ROS in neurodegenerative diseases (NDs)] (Biasiotto et al., 2019). However, traditional SWI faces a significant methodological barrier: its inability to distinguish between Fe^2+^ and Fe^3 +^ ions, which is crucial for a deeper understanding of the Fenton reaction. The deficiency in this qualitative capability represents a fundamental methodological limitation in current MRI-based research on iron-related oxidative stress in AD. It prevents researchers from determining whether the detected iron deposits are in an inert storage state (Fe^3 +^) or an active toxic state involved in catalyzing ROS generation (Fe^2+^), thereby severely restricting the depth of SWI results in elucidating pathological mechanisms and individualized risk assessment. Notably, only one pilot study has achieved this distinction by combining SWI with MRI relaxation measurement techniques, and *in vivo* research evidence is still lacking, remaining a research gap in this field (Dietrich et al., 2017). It should be emphasized that these techniques do not directly measure ROS, but instead indirectly indicate oxidative stress by detecting downstream indicators such as energy metabolism, iron deposition, or redox status.

While indirect methods emphasize the “result feedback” following ROS action, direct methods focus on the “real-time capture” of ROS molecules themselves. These two approaches can enhance ROS detection systems from different perspectives. In terms of direct angle, in AD research, to verify the hypothesis of high concentrations of ROS in the brain *in vivo* and noninvasively, and to provide evidence at both microscopic and macroscopic levels, researchers were inspired by “glow stick chemistry” and designed a novel near-infrared fluorescent (NIRF) imaging probe called 2-(4′-dimethylaminophenyl)-benzothiazolyl-derived cyanine dye (CRANAD-61) (Yang et al., 2017). The core innovation of this probe lies in its wavelength shift design: when reacting with ROS, its excitation and emission wavelengths undergo significant blue shifts, thereby enabling unique dual-channel ratio imaging and providing higher reliability for results. This allows it to distinguish “active” Aβ plaques surrounded by high ROS from “resting” plaques at the microscopic level (e.g., two-photon imaging) and to quantitatively measure the relative total ROS concentration in the brain of AD models at the macroscopic level (whole-body NIRF imaging). It successfully monitored the elevation of ROS levels with age (4–12 months) (Yang et al., 2017). However, despite the outstanding performance of CRANAD-61 in preclinical models, its clinical translation to humans faces core challenges, with the most prominent being the inherent tissue penetration depth limitation of NIRF, which is a major technical bottleneck for its application in deep brain imaging (Yang et al., 2019). To overcome this limitation, the research team innovatively combined CRANAD-61 with a strategy known as near-infrared fluorescence ophthalmic imaging (NIRFOI) (Yang et al., 2019). Leveraging the retina as the directly observable part of the central nervous system and the excellent light transmittance of the eyeball, NIRFOI provides a unique “brain window” for noninvasive monitoring of ROS and Aβ pathology associated with AD. Experimental evidence confirms that CRANAD-61’s ROS signal can be successfully detected in the eyes of AD model mice using NIRFOI (Yang et al., 2019). Therefore, a key direction for advancing the clinical translation of this technology in the future is to deepen research on such noninvasive combined imaging strategies to circumvent penetration depth limitations (Yang et al., 2019). From a therapeutic perspective, the potential of CRANAD-61 manifests across different stages of the AD disease course: in the early disease phase, it may serve as a screening tool and high-sensitivity biomarker for evaluating the immediate efficacy of disease-modifying therapies such as antioxidants (Yang et al., 2019; Yang et al., 2017); in the late disease phase, its ability to distinguish “active” plaques may enable precise monitoring of the direct pathological impact of targeted Aβ clearance therapies, thereby facilitating dynamic assessment of treatment responses (Yang et al., 2017).

In addition, we have found that a variety of neuroimaging methods provide an effective means for *in vivo* and noninvasive assessment of brain oxidative stress levels. These techniques complement each other and constitute a multi-dimensional research system. Some of the key technologies are described below. First, proton magnetic resonance spectroscopy imaging (^1^H-MRSI) can quantitatively detect low-molecular-weight antioxidants, especially glutathione (GSH) (Mandal et al., 2015; Mandal et al., 2019; Mischley et al., 2016). GSH plays a core role in antioxidant defense due to the reducing properties of its thiol group and is a key molecule for neurons to combat oxidative stress (Dwivedi et al., 2020). Its levels are significantly reduced in various NDs and have been widely recognized as a reliable biomarker. However, since the magnetic resonance spectral peaks of GSH often overlap with other metabolites, spectral editing techniques such as MEGA-PRESS are needed to achieve accurate separation and quantification (Shukla et al., 2020). The important value of this method lies in its ability to evaluate the dynamic changes in GSH or its precursor N-acetylcysteine (NAC) after supplementation therapy, thereby providing a basis for individualized clinical treatment strategies (Prasuhn et al., 2022). Currently, this technology primarily serves as a robust research tool, with its clinical value having been empirically validated in studies targeting the early stages of AD. For instance, a 2025 study demonstrated that in patients with mild cognitive impairment (MCI) of AD type, the levels of GSH measured in the frontal white matter via ^1^H-MRS were significantly associated with more severe vascular brain injury and cerebral atrophy (Detcheverry et al., 2025). However, one of the core bottlenecks hindering its clinical routine application lies in the challenges of technical measurement reproducibility. Studies have shown that the measurement accuracy of different metabolites (including GSH) using sequences such as MEGA-PRESS varies under different conditions, affecting the reliability of results and cross-center comparisons (Vidyasagar et al., 2024). Looking ahead, the key direction for advancing this technology is clearly focused on overcoming technical limitations. This includes developing faster imaging sequences and advanced reconstruction algorithms to address issues such as prolonged acquisition time and low SNR, as well as actively utilizing ultra-high field strengths (e.g., 7T) to fundamentally enhance detection efficiency and spectral resolution (Guo et al., 2025). The most promising direction lies in promoting multimodal image fusion, which involves correlating the unique metabolic information provided by ^1^H-MRS with the Aβ and tau pathological distribution shown by PET and imaging biomarkers reflecting CBF and function (Kara et al., 2026; Kara and Kantarci, 2024). This strategy is expected to more comprehensively elucidate the pathological mechanisms of AD and provide critical objective imaging biomarkers for dynamically evaluating the efficacy of targeted therapies.

Unlike the aforementioned evaluation of antioxidant capacity, PET directly reflects the degree of oxidative stress damage by employing the radioactive ligand copper-62 diacetyl-bis (N4-methylthiosemicarbazone) (^62^Cu-ATSM) as a specific molecular marker. Its detection principle is closely related to cellular biological mechanisms (Ikawa et al., 2020). Specifically, ^62^Cu-ATSM has unique physicochemical properties: when cells are in an overly reduced state (a typical accompanying phenomenon during oxidative stress), this tracer selectively accumulates within the cells (Ikawa et al., 2020). To verify the reliability of this principle, researchers conducted *in vitro* experiments. The results showed that in cell line models where ROS levels are abnormally elevated due to mitochondrial dysfunction, the retention of ^62^Cu-ATSM in cells is significantly increased (Donnelly et al., 2012; Yoshii et al., 2012). This result further confirms the association between the ligand and the oxidative stress state. Building upon the mechanisms confirmed *in vitro* studies, subsequent *in vivo* imaging research further validated the application potential of ^62^Cu-ATSM. Preclinical and preliminary clinical studies demonstrated that the radiotracer ^62^Cu-ATSM can be used for brain PET imaging in NDs such as PD and amyotrophic lateral sclerosis (ALS), with its signal intensity correlating with disease severity, preliminarily confirming its potential for *in vivo* visualization of oxidative stress-related pathology (Ikawa et al., 2020). However, this technology is currently primarily applied in research settings, and its translation into routine clinical diagnostics faces clear bottlenecks: the extremely short half-life of ^62^Cu (approximately 9.7 min) necessitates reliance on on-site radionuclide generators for labeling, significantly limiting its accessibility (Ng et al., 2014); additionally, existing human study samples typically have small sample sizes, restricting the generalizability of results. Future directions in this field include: exploring ^64^Cu-labeled ATSM with longer half-lives to expand its applications in targeted radionuclide therapy (i.e., “diagnosis and treatment integration”) (Hong et al., 2025; Lopci et al., 2016); promoting its integration with multimodal techniques such as MRI to obtain more comprehensive pathological information (Simon et al., 2025; Zubair et al., 2025); and developing novel probes with higher selectivity (Hong et al., 2025).

As a pivotal breakthrough in the field of molecular imaging, the newly developed PET probe 2-deoxy-2-[^18^F]fluoro-α-D-arabinofuranosylvinyluracil (^18^F-FEDV) can be directly utilized for *in vivo* oxidative stress imaging (Wilde et al., 2025). The probe exhibits broad-spectrum reactivity with reactive oxygen and nitrogen species (RONS), excellent blood-brain barrier (BBB) penetration, and high stability. It specifically detects elevated levels of oxidative stress in various mouse models (intrastriatal injection of sodium nitroprusside (SNP), middle cerebral artery photothrombotic stroke model, and P301S tauopathy AD model). In contrast, clinically commonly used [^18^F]FDG (glucose metabolism imaging) and another probe for detecting innate immune activation, [^18^F]FN, fail to effectively differentiate these models. During the study, this probe also employed dynamic PET imaging combined with parametric mapping to calculate tracer uptake rate (Ki), significantly enhancing detection sensitivity. This method can detect elevated oxidative stress as early as 4 h after stroke, while traditional static analysis using standardized uptake values (SUV) cannot detect significant differences at this time (Wilde et al., 2025). However, it is essential to objectively recognize that ^18^F-FEDV, as an emerging PET imaging technology, is currently still primarily in the preclinical research stage (Wilde et al., 2025). Although it has successfully achieved specific imaging of oxidative stress in the central nervous system in mouse models of AD and stroke (Wilde et al., 2025), there remain clear challenges in advancing toward routine clinical application. The primary challenge is the bottleneck in automated production. Studies have shown that the yield of manually synthesized radiopharmaceuticals is approximately 12%, but this significantly decreases to 3–5% when transferred to commercial automated synthesis modules (Ghosh et al., 2025). Secondly, the field lacks a unified standard procedure for imaging and quantification, which hinders the direct comparison and interpretation of different research results (Waters et al., 2018). Looking ahead, the development of this technology should focus on several key directions: optimizing automated synthesis protocols to achieve stable and high-yield preparation (Ghosh et al., 2025); rigorously validating the correlation between its imaging signals and classical biomarkers of oxidative damage in more disease models to establish standardized methods (Waters et al., 2018); and ultimately, leveraging its unique advantages derived from approved drugs to explore its potential in precision medicine, integrating diagnosis and therapeutic evaluation (Wilde et al., 2025).

It is noteworthy that ^18^F-ROStrace is the first PET tracer capable of directly measuring ROS in the brain, with its development predating ^18^F-FEDV (Hou et al., 2018). Its core functionality involves noninvasive quantitative imaging of oxidative stress levels by inducing intracellular retention following ROS oxidation (Gallagher et al., 2025; Park et al., 2026). The tracer is designed based on dihydroethidium (DHE) derivatives, exhibiting high selectivity for superoxide anions but low background uptake in normal tissues (Gallagher et al., 2025; Park et al., 2026). Both *in vitro* and *in vivo* experiments demonstrate that ^18^F-ROStrace uptake signals accurately reflect intracellular ROS levels and exhibit strong consistency with biological and pathological indicators related to oxidative stress (Gallagher et al., 2025; Park et al., 2026). The technological advantage lies in enabling direct, quantitative, and dynamic visualization of oxidative stress *in vivo* (Gallagher et al., 2025; Park et al., 2026). However, its limitations are also evident: first, the tracer reflects total intracellular ROS levels without distinguishing between mitochondrial and cytoplasmic ROS sources (Gallagher et al., 2025; Park et al., 2026); second, *in vivo* imaging uptake may be influenced by blood perfusion and systemic inflammatory states, leading to inter-animal variability potentially attributable to differences in drug metabolism rates rather than intrinsic tissue oxidative stress levels (Park et al., 2026); additionally, signal normalization requires selection of appropriate reference brain regions (e.g., corpus callosum) for differentiation between disease-related and nonspecific ROS signals (Gallagher et al., 2025). In terms of disease applications, current evidence supports its potential in AD, as it has been successfully applied in APP/PS1 and tau PS19 mice to detect oxidative stress and predict pathology progression, yet data are derived from preclinical animal models and have not yet entered human studies (Gallagher et al., 2025; Park et al., 2026). Moreover, the PS19 model exhibits phenotypic drift, with varying pathological severity and age-related patterns among individuals, which may affect the interpretation of imaging results (Gallagher et al., 2025). Future research directions can encompass multiple dimensions: first, this tracer requires validation across more diverse disease models, such as PD; second, longitudinal PET imaging can be integrated to track the dynamic evolution of oxidative stress during disease progression; additionally, combined use of mitochondrial-specific probes can further delineate the specific sources of ROS; finally, this technology can be incorporated into antioxidant or anti-inflammatory intervention studies, where changes in tracer signals before and after treatment can be compared to evaluate the efficacy of targeted oxidative stress therapies (Gallagher et al., 2024; Gallagher et al., 2025; Park et al., 2026; Zhu et al., 2024).

This section elucidates the key mechanisms by which oxidative stress and iron metabolism disorders induce mitochondrial dysfunction in AD, further discusses how the aforementioned imaging techniques can assess this pathological process *in vivo* from multiple dimensions, and analyzes the characteristics and limitations of each technique. In terms of current applications and advantages/disadvantages, QSM demonstrates elevated iron concentrations in the hippocampus of AD patients, indicating a pathological environment conducive to ROS generation, while posing no radiation risk. However, it requires high magnetic field homogeneity and remains in the optimization phase (Onukwufor et al., 2022; Stone and Blockley, 2017). The QSM-quantitative blood oxygen level-dependent fusion QQ model enables more comprehensive assessment of oxygen metabolism but is also in the developmental stage (Biondetti et al., 2023). ^1^H-MRSI quantitatively detects the key antioxidant GSH, whose reduced levels serve as reliable biomarkers. However, measurement reproducibility is affected by spectral peak overlap and technical variations (Mandal et al., 2015; Shukla et al., 2020). Superparamagnetic ^13^C-MRI employs tracers such as [^1−13^C]pyruvate to indirectly reflect redox status through metabolite ratios, but demands high equipment standards and complex tracer preparation (Sharma et al., 2024). QUEST-MRI indirectly reflects the overall oxidative state by measuring changes in relaxation rates after antioxidant treatment, without the need for exogenous contrast agents. However, it lacks the ability to identify specific ROS and has not been systematically applied in AD patients (Berkowitz, 2018; Prasuhn et al., 2022). PET with the radioactive ligand ^62^Cu-ATSM can selectively accumulate in hyperreduced cells for imaging oxidative stress, but it’s extremely short half-life limits clinical accessibility (Ikawa et al., 2020). Emerging direct detection probes exhibit higher specificity, such as NIRF imaging probe CRANAD-61, which can specifically image ROS and differentiate plaque activity in AD models, but faces clinical translation bottlenecks due to tissue penetration depth (Ashleigh et al., 2023). Although the novel PET probe ^18^F-FEDV enables direct *in vivo* imaging and quantitative analysis of oxidative stress in the central nervous system, it faces challenges such as low automated synthesis yield and lack of unified quantitative standards (Wilde et al., 2025). Complementing this, PET probe ^18^F-ROStrace, the first tracer enabling direct *in vivo* ROS imaging via ROS-induced intracellular retention, offers high selectivity and low background uptake but is limited by its inability to distinguish ROS sources, potential perfusion confounding, and lack of human validation (Gallagher et al., 2025; Park et al., 2026). Additionally, iron-sensitive MRI techniques (such as SWI) indirectly reflect oxidative stress by detecting iron deposition, but traditional SWI cannot distinguish between Fe^2+^ involved in the Fenton reaction and the relatively inert Fe^3 +^, which represents a fundamental methodological limitation (Biasiotto et al., 2019; Dietrich et al., 2017). However, several research gaps remain. For instance, existing indirect techniques are prone to interference and lack specificity, while the safety and quantitative standardization of direct probes in humans require urgent validation. Most critically, there is an acute shortage of prospective imaging studies capable of longitudinally and dynamically tracking ROS levels and Aβ and tau pathological interactions throughout the entire AD disease course (Green et al., 2024). Future efforts should focus on advancing the clinical translation and standardization of direct probes, optimizing the specificity and reliability of indirect techniques, and integrating multimodal imaging to comprehensively elucidate the dynamic role of oxidative stress in AD.

#### 3.1.3 Mitochondrial ultrastructure and dynamic abnormalities

In AD, mitochondrial quality control imbalance is a crucial pathological feature, primarily characterized by two core mechanisms: dysfunction of mitophagy and disruption of mitochondrial dynamics. Among these, mitophagy dysfunction plays a central role in mediating AD-related mitochondrial dysfunction (Wong et al., 2026). The pathological characteristics and molecular regulatory mechanisms underlying this dysfunction can be analyzed from several perspectives. From a phenotypic standpoint, extensive research has demonstrated significant autophagy dysfunction in the brain tissue of AD patients. This dysfunction is characterized by three key indicators: 1) a markedly elevated ratio of LC3-II (lipidated form of autophagy-associated protein LC3) to LC3-I (unmodified form) (LC3-II/I), 2) abnormal accumulation of autophagy substrate protein p62, and 3) significantly reduced co-localization between mitochondria and lysosomes (Wang et al., 2019). These autophagy impairment indicators directly suggest impaired clearance of damaged mitochondria in the AD brain. In-depth analysis of its molecular regulatory mechanisms reveals that this pathological phenomenon is closely associated with abnormalities in the PINK1/Parkin pathway. Studies have shown that during the pathological progression of AD, the expression of the PINK1/Parkin pathway exhibits significant stage-specific changes. In early-stage AD (Braak stages II-III), the expression level of PINK1, a key molecule in this pathway, was significantly elevated. This change is considered to be a compensatory stress response to early mitochondrial damage (Goiran et al., 2018). This upregulation attempts to activate the PINK1/Parkin pathway for the clearance of dysfunctional mitochondria. However, this compensatory mechanism fails to activate effective mitophagy. Evidence shows that despite elevated PINK1 levels, mitochondrial quality markers [such as translocase of the outer mitochondrial membrane 20 kDa homolog (TOMM20)] and mtDNA copy numbers also increased, indicating that damaged mitochondria actually accumulated rather than decreased. At the late stages of AD (Braak stages V-VI), not only is PINK1 expression downregulated, but the expression of another key molecule, Parkin, is also significantly disrupted (Martín-Maestro et al., 2016). Specifically, the downregulation of PINK1 may be associated with persistent mitochondrial dysfunction, while Parkin’s expression dysregulation manifests as inconsistent findings across studies (Ma et al., 2016; Wang et al., 2018; Wang et al., 2019). This inconsistency may be attributed to the inherent complexity of the PINK1/Parkin pathway. Recent studies have demonstrated that PINK1 exhibits significant regional specificity and subcellular distribution differences in the brain, with low endogenous expression levels and high sensitivity to detection techniques (Liu et al., 2025). Furthermore, the function of PINK1 extends beyond mitophagy, involving the regulation of multiple cellular homeostasis (Yoboue and Valente, 2020). Consequently, significant variations in the molecular expression patterns of this pathway may be observed across different pathological stages, brain regions, or detection methods. This heterogeneity in expression patterns reflects the severe functional impairment of the pathway in advanced disease stages. This phenomenon may stem from deeper mechanisms: on one hand, Parkin expression may undergo compensatory upregulation under specific conditions; on the other hand, the pathway’s function may be severely impaired. For instance, Parkin may abnormally interact with pathological p-tau, hindering their normal transport to damaged mitochondria and preventing ubiquitination labeling (Cummins et al., 2019). Ultimately, both the initiation and execution phases of the mitophagy pathway are obstructed, leading to the accumulation of defective mitochondria within neurons and accelerating disease progression. In subsequent analyses, we also observed that this stage-specific dysfunction essentially reflects the collapse of the compensatory autophagy mechanism in AD brains: In early stages, the blocking effect of tau/Aβ causes “ineffective activation” of autophagy initiation; in late stages, dysregulation of key pathway molecules leads to “functional failure” of autophagy signaling (Cummins et al., 2019). This process directly results in excessive accumulation of damaged mitochondria within cells, significantly reduced clearance efficiency, and further amplification of AD’s pathological damage effects through activation of the ROS-mtDNA-inflammatory response axis and cellular energy metabolism crisis (Li et al., 2022; Mary et al., 2023). Therefore, future research should focus on standardizing sampling brain regions, disease staging, and detection techniques to precisely elucidate the dynamic changes in the PINK1/Parkin pathway during the pathogenesis and progression of AD. Furthermore, various AD-related molecules can regulate mitophagy through different mechanisms, exacerbating autophagy dysfunction. First, full-length p-tau or its pathogenic mutants (e.g., P301L) can directly block autophagy initiation by inhibiting Parkin’s translocation to mitochondria. Second, while Aβ_1–42_ oligomers induce mitophagy initiation, they block subsequent degradation processes, ultimately leading to accumulation of LC3-II and p62 within cells (Gou et al., 2021). Third, monomeric Aβ can impair lysosome-mediated autophagy degradation by suppressing key lysosomal hydrolases (e.g., cathepsin D). Fourth, Apolipoprotein E ε4 allele (APOE4) genotype competitively binds to the autophagy regulatory transcription factor transcription factor EB (TFEB), downregulating expression levels of autophagy-related genes such as p62 and LC3B, thereby indirectly affecting mitophagy function (Mary et al., 2023).

Beyond the critical impairment of mitochondrial quality control mechanisms caused by autophagy defects in AD, mitochondrial dynamics imbalance also plays a pivotal role in AD’s pathological progression. This dynamic equilibrium primarily depends on the coordinated regulation of fission and fusion processes, which are essential for maintaining mitochondrial structural integrity, functional stability, and cellular adaptability. Under normal physiological conditions, mitochondrial fission is predominantly regulated by the fission proteins dynamin-related protein 1 (Drp1) and fission-associated protein (Fis1). Upon activation, Drp1 translocates from the cytoplasm to the mitochondrial outer membrane, initiating fission (Gowda et al., 2022; Monzio Compagnoni et al., 2020). In contrast, mitochondrial fusion is regulated by the fusion proteins mitofusin 1 (Mfn1), Mfn2, and optic atrophy 1 (OPA1), with Mfn1/2 mediating outer membrane fusion and OPA1 regulating inner membrane fusion, collectively preserving mitochondrial network continuity (Mary et al., 2023). However, in AD, this balance between fission and fusion is significantly disrupted, leading to an “overactive fission-suppressed fusion” imbalance. Specifically, Aβ and phosphorylated p-tau directly interact with Drp1, significantly enhancing its activity and promoting excessive mitochondrial fission. Concurrently, studies have demonstrated markedly elevated expression levels of Fis1 in AD patients’ brain tissue, further exacerbating the abnormal activation of fission processes. On the other hand, the expression levels of AD brain fusion-related proteins (Mfn1, Mfn2, and OPA1) are significantly downregulated, leading to impaired mitochondrial fusion function. Notably, reduced expression of the fusion protein OPA1 not only directly inhibits mitochondrial fusion but also causes excessive accumulation of fragmented mitochondria within cells, ultimately accelerating neuronal apoptosis (Li et al., 2022). Additionally, various pathological factors can exacerbate mitochondrial dynamic imbalance through regulation of key molecules. For instance, iron ion dysregulation (such as Fe^3 +^ accumulation) can activate calmodulin-dependent phosphatase (CDPP), inducing dephosphorylation of Drp1 and further enhancing its mediated mitochondrial fission activity (Onukwufor et al., 2022). It is noteworthy that while microRNA miR-455-3p has been proven to be induced by Aβ feedback and possesses regulatory effects promoting mitochondrial fusion, its expression levels exhibit significant abnormalities in AD pathological environments. This prevents miR-455-3p from properly performing its fusion regulation function, thereby exacerbating mitochondrial dynamic imbalance (Gowda et al., 2022).

Dysregulation of mitophagy and dynamic balance collectively leads to mitochondrial dysfunction, which in turn accelerates the pathological progression of AD. The accumulation of damaged mitochondria not only increases the production of ROS but also further damages mtDNA and respiratory chain proteins, forming a vicious cycle (Gowda et al., 2022; Onukwufor et al., 2022). Moreover, mitochondrial dysfunction can also affect the energy supply to neurons, leading to reduced neurotransmitter release, impaired synaptic transmission, and ultimately resulting in cognitive impairment and memory decline (Han et al., 2020). Therefore, a deeper understanding of the role of mitochondrial quality control mechanisms in AD is of significant importance for the development of new therapeutic strategies. Future research can focus on how to regulate the PINK1/Parkin pathway, restore mitochondrial dynamics balance, and enhance mitophagy function, aiming to provide new targets and methods for the treatment of AD.

Therefore, in-depth structural analysis of the aforementioned ultrastructural abnormalities has become a critical research focus. Structural studies employing high-resolution electron microscopy (EM) techniques, particularly three-dimensional electron microscopy (3DEM), can reveal ultrastructural abnormalities in AD, such as mitochondrial cristae membrane disruption and structural fragmentation (Ju et al., 2023). 3DEM encompasses three-dimensional structural analysis techniques: EM tomography, serial block-face electron microscopy (SBEM), and focused ion beam scanning electron microscopy (FIB-SEM). These methods aim to investigate the morphology, distribution, and structural changes of cellular and subcellular structures (e.g., mitochondria) in three-dimensional space. EM tomography, with its highest resolution (approximately 5 nm), provides exceptional clarity for fine structures like mitochondrial cristae membranes, establishing it as the gold standard for cristae architecture analysis. However, its limited imaging volume makes it challenging to capture complete mitochondrial networks, restricting high-throughput applications (Choi et al., 2020). In contrast, SBEM excels at acquiring large tissue volumes (especially along the Z-axis), enabling high-throughput analysis of mitochondrial networks (e.g., over a thousand mitochondria per volume) through counting and length measurements. However, its lower resolution (7–10 nm in X/Y planes and 40–80 nm in Z-axis) fails to resolve cristae membrane details adequately (Colasuonno et al., 2017). To address this resolution bottleneck, FIB-SEM emerges as a promising compromise, maintaining comparable volumetric acquisition capabilities to SBEM while delivering more clearly defined cristae membrane images. It is crucial to note that these technologies have evolved beyond being mere tools for pathophysiological description to become pivotal means for evaluating potential therapeutic strategies. For instance, in the AD mouse model, researchers utilized SBEM combined with three-dimensional reconstruction techniques to precisely assess changes in mitochondria-associated endoplasmic reticulum membranes (MAMs), identifying specific mitochondrial-endoplasmic reticulum membrane stabilizers that modulate amyloid protein production and influence mitochondrial axonal transport velocity (Zellmer et al., 2025). Another study demonstrated through 3DEM technology that an experimental drug targeting mitochondrial complexes effectively restored synaptic density in the hippocampus of AD mice (Keller et al., 2024). Although these techniques exhibit irreplaceable value in elucidating AD mechanisms and evaluating therapeutic outcomes, their current applications remain confined to preclinical research (Keller et al., 2024; Panes et al., 2023). The primary bottleneck in clinical translation lies in the highly complex and time-consuming sample preparation and data analysis workflows, coupled with the lack of standardized operational and analytical procedures (Panes et al., 2023; Zellmer et al., 2025). In the future, automated image analysis algorithms integrated with AI will be pivotal in enhancing 3DEM data processing efficiency and promoting broader applications in AD therapeutic target discovery and efficacy evaluation (Panes et al., 2023).

This section focuses on mitochondrial quality control dysregulation in AD, including autophagy dysfunction and fusion dynamics imbalance. High-resolution structural imaging techniques provide unique insights into these processes. In terms of current applications and limitations, high-resolution EM, particularly 3DEM plays an irreplaceable role in investigating ultrastructural abnormalities such as mitochondrial cristae membrane disruption, structural fragmentation, and autophagosome formation (Ju et al., 2023). 3DEM encompasses electron tomography (with the highest resolution, approximately 5 nm), SBEM (high-throughput but lower resolution), and FIB-SEM (a compromise approach) (Choi et al., 2020; Colasuonno et al., 2017). These techniques have evolved beyond being mere tools for pathological description to become critical for evaluating potential therapeutic strategies in preclinical models. However, their application remains strictly limited to preclinical research, with the primary bottleneck for clinical translation being the highly complex and time-consuming sample preparation and data analysis workflows, as well as the lack of standardized protocols (Colasuonno et al., 2017). In terms of research gaps and future directions, there is currently a lack of clinical imaging technologies capable of visualizing mitochondrial dynamic processes (e.g., autophagic flux, fusion events) *in vivo* and noninvasively (Haessler et al., 2024; Niederschweiberer et al., 2021). A key future direction involves developing AI-integrated automated image analysis algorithms to significantly enhance processing efficiency for complex data like 3DEM, thereby expanding their applications in AD treatment target discovery and efficacy evaluation (Shawa and Phiri, 2025). Concurrently, exploring the correlation between biophysical biomarkers derived from ultrastructural studies and macroscopic imaging represents a critical frontier in bridging microscopic pathology with macroscopic phenotypes (Yu et al., 2025).

Defects in mitochondrial bioenergetic metabolism, oxidative stress, and dynamic imbalance not only directly impair neuronal function but may also interact with Aβ protein and p-tau to form a pathological cascade that accelerates the progression of AD. The specific mechanisms are elaborated in the following section.

### 3.2 Mitochondrial interaction with Aβ and tau

#### 3.2.1 core pathological mechanism: oxidative stress-driven vicious cycle

In AD, mitochondrial dysfunction and Aβ and tau pathology are not isolated but form a vicious cycle that exacerbates each other, with oxidative stress serving as the core driver of this cycle (Monzio Compagnoni et al., 2020; Swerdlow, 2018; Sun B. et al., 2025; Wasim, 2025).

In this cycle, direct mitochondrial damage caused by Aβ oligomers is a critical step in triggering neuronal dysfunction. Firstly, the deposition of Aβ plaques affects mitochondrial membrane potential; specifically, Aβ oligomers can directly damage the mitochondrial membrane, leading to a decrease in mitochondrial membrane potential (Monzio Compagnoni et al., 2020; Swerdlow, 2018). Additionally, Aβ induces mitochondrial membrane depolarization by inhibiting the activity of cytochrome c oxidase (COX, complex IV) in the ETC, resulting in a persistent reduction of membrane potential and subsequent mitochondrial dysfunction (Monzio Compagnoni et al., 2020; Swerdlow, 2018). Aβ also binds to metal ions (e.g., Cu^2+^/Fe^2+^), promoting the excessive generation of ROS through the Fenton reaction, further damaging mtDNA and respiratory chain proteins, and exacerbating mitochondrial oxidative stress (Gowda et al., 2022; Yu et al., 2024). Furthermore, Aβ*1*-42 oligomers can bind to mitochondrial proteins Aβ-binding alcohol dehydrogenase (ABAD) and cyclophilin D (CypD), inducing oxidative stress and apoptosis (Swerdlow, 2018). Beyond their effects on oxidative stress and membrane potential, amyloid-β precursor protein (AβPP) can also block mitochondrial protein import channels, further reducing COX activity (Swerdlow, 2018).

In addition to the aforementioned direct injury pathways, the accumulation of Aβ plaques can also trigger mitochondria-dependent apoptosis through another mechanism by disrupting calcium homeostasis. Specifically, mitochondrial calcium overload induces the opening of the mitochondrial permeability transition pore (mPTP), thereby activating neuronal apoptosis (Swerdlow, 2018). This process is primarily mediated by the mitochondrial calcium uniporter (MCU). When matrix calcium overload exceeds the physiological threshold, the sustained opening of mPTP results in mitochondrial membrane potential collapse and the release of pro-apoptotic factors (Matuz-Mares et al., 2022).

On the other hand, excessive phosphorylation of p-tau can also impair mitochondrial function. Specifically, overphosphorylated p-tau disrupts axonal mitochondrial transport, leading to a reduction in the number of mitochondria at synaptic sites and consequently causing axonal mitochondrial depletion (Monzio Compagnoni et al., 2020). This mitochondrial transport disorder not only affects neuronal energy supply but also disrupts normal mitochondrial fission and fusion mechanisms, exacerbating mitochondrial fragmentation (Mary et al., 2023). Meanwhile, in the brain tissue of AD patients, the nuclear-encoded genes for mitochondrial complexes I–V are significantly downregulated, which further aggravate mitochondrial energy metabolism defects.

Aβ and p-tau not only independently damage mitochondria but also induce oxidative stress, forming a synergistic vicious cycle that serves as the core driving force in the progression of AD. On the one hand, Aβ oligomers directly target mitochondria by inhibiting the function of the ETC complex, disrupting membrane potential homeostasis, and inducing mitochondrial calcium overload, thereby directly triggering ROS bursts. On the other hand, hyperphosphorylated p-tau hinder mitochondrial axonal transport, leading to energy depletion at synaptic terminals and reduced local ROS clearance capacity (Moawad et al., 2025). These ROS generated from different sources do not exist in isolation; they further attack mtDNA and respiratory chain proteins, exacerbating energy metabolism defects. Simultaneously, ROS-activated specific signaling pathways [e.g., inhibition of protein phosphatase 2A (PP2A)/activation of glycogen synthase kinase-3 beta (GSK3β)] provide positive feedback to promote abnormal p-tau phosphorylation and Aβ generation, thereby forming a self-reinforcing pathological closed loop (Hroudová and Fišar, 2025).

This “oxidative stress vicious cycle” further impairs mitochondrial quality control mechanisms. On the one hand, Aβ1-42 oligomers can induce the initiation of mitophagy but block subsequent degradation, leading to the accumulation of LC3-II and p62 (Mary et al., 2023); on the other hand, abnormal accumulation of p-tau interferes with mitophagy, exacerbating the accumulation of damaged mitochondria. The aforementioned mitophagy defects primarily manifest as damage to the PINK1/Parkin pathway mentioned in the previous section. Specifically, Parkin deficiency worsens mitophagy defects caused by p-tau accumulation, while overexpression of PINK1 alleviates oxidative stress and reduces Aβ levels by activating the OPTN/NDP52 receptor (Liu et al., 2023; Moawad et al., 2025).

#### 3.2.2 Multidimensional validation of neuroimaging: from molecules to networks

The pathological basis of the aforementioned “oxygenative stress vicious cycle” provides critical intervention targets for neuroimaging studies. Imaging not only confirms the interaction among Aβ, tau, and mitochondria but also possesses the capability to dynamically monitor its key hub—oxidative stress.

Firstly, molecular imaging can directly visualize pathological proteins and energy metabolism. For example, PET plays an important role in the study of AD pathological mechanisms. Among these, PET tracers targeting Aβ [such as [^18^F]florbetapir (^18^F-AV45)] have been widely used in clinical practice and research to detect the deposition of Aβ plaques in the brains of AD patients, enabling early diagnosis and differential diagnosis of the disease (Clark et al., 2011; Sabri et al., 2015). From the perspective of clinical application and acceptance, amyloid PET imaging has established clear clinical guidelines and has become a key step in screening patients before disease-modifying therapy. International guidelines have explicitly defined the ‘appropriate use’ criteria for specific clinical scenarios (such as evaluating mild cognitive impairment or dementia of unknown cause), and its value and role in clinical decision-making are becoming increasingly clear.

The phase of capacity depletion in this vicious cycle has been previously discussed in the context of mitochondrial energy metabolism disorders. For instance, section 3.1.1 states that, ^18^F-BCPP-EF, as a novel tracer, targets complex I in the mitochondrial ETC. Preclinical studies have demonstrated its potential in evaluating cerebral energy metabolism status, which may be associated with the oxidative stress mechanism in AD (Tsukada et al., 2014). This technology has entered the early clinical research phase and has shown promise as a noninvasive biomarker in diseases such as Friedreich’s ataxia (FRDA). A pivotal animal model study in 2021 provided direct evidence for this direction: using dual-tracer PET imaging with ^18^F-BCPP-EF and carbon-11 Pittsburgh compound-B (^11^C-PiB, an Aβ tracer), researchers observed for the first time in animal models of early-stage AD (mild cognitive impairment phase) a significant negative correlation between decreased mitochondrial complex I availability in the brain and increased Aβ deposition *in vivo* (Tsukada et al., 2014). This finding provides crucial *in vivo* imaging evidence for the hypothesis that “Aβ pathology and mitochondrial energy depletion coexist and are interrelated in early-stage AD.”

Additionally, functional and structural imaging further supplement multidimensional information by revealing network effects and ultrastructural damage. In animal models, high-resolution functional imaging revealed the spatiotemporal evolution of the vicious cycle. In APP[SAA/+]-knock-in AD mouse models, two-photon fluorescence lifetime imaging (^2^P-FLIM) confirmed that by 4 months of age—when only soluble Aβ was present and no amyloid plaques were observed—the respiratory response of mitochondria to nutritional stimuli had been lost (abnormal NADH metabolism). Simultaneously, multi-parametric photoacoustic microscopy (MP-PAM) demonstrated cortical vascular oxygen consumption defects, confirming that mitochondrial dysfunction preceded Aβ deposition (Norambuena et al., 2024).

In the pathology of p-tau, diffusion tensor imaging (DTI) detects white matter ultrastructural damage through parameters such as fractional anisotropy (FA) and mean diffusivity (MD), confirming that p-tau-mediated mitochondrial transport dysfunction can disrupt white matter networks, leading to cognitive and gait impairments (Griffiths and Grant, 2023; Jett et al., 2023).

At the neural network level, resting-state functional magnetic resonance imaging (rs-fMRI) reveals abnormalities in the DMN, particularly in the medial temporal-hippocampal connection. These abnormalities are highly consistent with the neural network disruption caused by Aβ and p-tau-induced mitochondrial dysfunction (Griffiths and Grant, 2023; Jett et al., 2023). Notably, deep learning models integrating fMRI and DTI data (such as convolutional neural networks and graph neural networks) can improve early AD diagnosis accuracy achieving accuracies of 70–98% (Warren and Moustafa, 2023), highlighting the critical role of multimodal imaging in analyzing the “Aβ and tau-mitochondria-neural network” causal chain.

#### 3.2.3 Disputes over chronological order: the complexity of causality

The aforementioned evidence clearly delineates the vicious cycle of interwoven and mutually exacerbating interactions between Aβ, p-tau, and mitochondrial dysfunction (Monzio Compagnoni et al., 2020; Swerdlow, 2018). However, a fundamental question arises: where does this cycle originate? The temporal sequence and causal direction between mitochondrial dysfunction and Aβ and tau pathology remain controversial in current research, giving rise to two primary hypotheses.

Mechanistically, mtDNA mutations impair the function of respiratory chain complexes, leading to increased oxidative stress and reduced ATP production. These mitochondrial deficits, in turn, create a cellular environment conducive to Aβ generation and tau hyperphosphorylation, suggesting that mitochondrial dysfunction may occur prior to—and potentially drive—the pathological changes of Aβ and tau (Monzio Compagnoni et al., 2020). Specifically, mtDNA is highly sensitive to oxidative damage, and its cumulative mutations can lead to defects in the ETC, thereby promoting Aβ generation and tau phosphorylation, which constitutes the initial step of a vicious cycle (Chaturvedi and Flint Beal, 2013; Fišar, 2022; Lakatos et al., 2010). More importantly, high-resolution functional imaging in APP[SAA/+]-knock-in AD mouse models has confirmed that the loss of mitochondrial respiratory function occurs during the soluble Aβ phase and significantly precedes amyloid plaque deposition (Norambuena et al., 2024), providing robust dynamic imaging evidence for the “mitochondrial first” hypothesis.

In contrast, another school of research emphasizes that Aβ oligomers and phosphorylated p-tau can directly attack mitochondria, inducing their functional collapse and thereby initiating or amplifying subsequent degenerative processes (Monzio Compagnoni et al., 2020; Swerdlow, 2018). Three transgenic mouse models have demonstrated that the combined effect of Aβ and p-tau significantly exacerbates mitochondrial damage and neurodegenerative pathology (Monzio Compagnoni et al., 2020).

This ambiguity in causality precisely reveals the systemic and complex nature of the AD pathological mechanism: mitochondrial dysfunction and Aβ and tau pathology are likely not a simple linear causal relationship, but rather a symbiotic process of mutual triggering and synergistic progression across different disease stages (Ashleigh et al., 2023). Mitochondrial dysfunction in the AD pathogenesis may serve as both an initial event and a consequence of other pathological processes (Monzio Compagnoni et al., 2020).

#### 3.2.4 Research limitations and future directions

Although the aforementioned evidence system delineates the vicious cycle of Aβ and tau-mitochondrial interactions, and neuroimaging has made significant progress in validating and quantifying this mechanism, it must be acknowledged that the current research landscape remains fragmented, and this review also has certain limitations in elucidating the interactions among the three.

First, the imbalance in evidence hierarchy. Most imaging studies (e.g., ^18^F-BCPP-EF PET) remain at the preclinical stage, with their sensitivity and specificity in humans yet to be validated through large-scale clinical trials. Many molecular details regarding the interaction between Aβ and tau and mitochondria (e.g., the specific flux of AβPP blocking protein import channels, the regulatory mechanisms of the PINK1-Parkin pathway in humans) still primarily rely on *in vitro* experiments or animal models, lacking robust *in vivo* evidence (Hroudová and Fišar, 2025). This gap between mechanistic understanding and imaging translation constitutes a potential source of inaccuracy in current comprehension.

Second, the dynamic evolution is inadequately characterized. Although the “vicious cycle” model helps capture the core driving force of AD progression, this model represents a necessary simplification of a highly complex pathological process involving multiple feedback loops, diverse cell types, and significant spatiotemporal heterogeneity (Fišar, 2022). Current literature lacks clarity on the weight variations of different pathological events across disease stages (e.g., is oxidative stress dominant in the early stage, or mitophagy defects in the late stage? Does Aβ oligomer first attack mitochondria, or does mitochondrial dysfunction precede it?). This results in our understanding of the cycle remaining static rather than dynamically evolving.

Third, the lack of multi-target synchronous imaging technology. Currently, there is no technique capable of simultaneously and directly visualizing the interaction between mitochondrial function and Aβ and tau pathology *in vivo*. Although carbon-11 acetate (^11^C-acetate) can reflect cellular oxidative metabolic levels, its current applications are primarily focused on tumor imaging, with relatively limited use in the nervous system.

Therefore, future research frontiers lie in developing “multi-target” tracers or deeply integrated multimodal imaging protocols (e.g., mitochondrial function PET + Aβ-PET + tau-PET + fMRI) to noninvasively map the complete pathological atlas of “Aβ and tau-mitochondria-neural network” in a single examination (Bell et al., 2021; Murphy and Hartley, 2018; Steele et al., 2017). The integration of multimodal imaging across different pathological dimensions, or the use of “multi-target” tracers capable of reflecting multiple biological processes in a single scan, is becoming a critical frontier for improving the accuracy of AD diagnosis, elucidating the complex mechanisms of the disease, and evaluating the efficacy of novel therapies (Fišar et al., 2019; Mishra et al., 2019; Murphy and Hartley, 2018). Therapeutic strategies targeting mitochondrial dysfunction (e.g., enhancing mitophagy, modulating kinetic equilibrium, improving energy metabolism, and alleviating oxidative stress) may provide new directions for AD treatment, potentially breaking the vicious cycle of progression through multiple mechanisms (Bhatti et al., 2023; Ye et al., 2015). Future research should not be confined to identifying a single “primary etiology” but should focus on dismantling this holistic pathological network, while requiring more longitudinal studies to elucidate the dynamic weights of different pathological events in disease progression.

This section delves into the vicious cycle of mutual exacerbation between mitochondria and Aβ/tau pathology (Monzio Compagnoni et al., 2020; Swerdlow, 2018). Neuroimaging not only confirms the existence of this interaction but also plays a pivotal role in elucidating its spatiotemporal sequence and network effects. Among these, amyloid PET (e.g., ^18^F-AV45) has been clinically utilized for detecting Aβ deposition and has established clear clinical guidelines, though it remains limited in nonspecific binding to white matter and low-sensitivity detection at low loads (Clark et al., 2011; Sabri et al., 2015). In animal models, ^2^P-FLIM and MP-PAM have provided dynamic imaging evidence that mitochondrial respiratory dysfunction occurs during the soluble Aβ phase, preceding plaque deposition (Norambuena et al., 2024). In human studies, DTI detects tau-mediated white matter damage through parameters such as FA (Griffiths and Grant, 2023; Jett et al., 2023); rs-fMRI demonstrates that DMN abnormalities are consistent with neural network disruption caused by Aβ/tau-induced mitochondrial dysfunction (Jett et al., 2023; Warren and Moustafa, 2023). Deep learning models integrating fMRI and DTI data can improve the accuracy of early diagnosis (Warren and Moustafa, 2023). The combined imaging of the mitochondrial complex I-targeting PET tracer ^18^F-BCPP-EF and the Aβ tracer provides *in vivo* evidence for the coexistence of “Aβ pathology and mitochondrial energy failure in the early stages of AD” in animal models (Tsukada et al., 2014). However, there remains a research gap, as no technology currently exists to simultaneously and directly visualize the interaction between mitochondrial function and Aβ/tau pathology *in vivo*. Future research frontiers include developing “multi-target” tracers or deeply integrated multimodal imaging protocols (e.g., mitochondrial function PET + Aβ-PET + tau-PET + fMRI) to noninvasively map the complete pathological atlas of “Aβ/tau-mitochondria-neural network” in a single examination, thereby precisely guiding personalized treatment and efficacy evaluation.

In addition to the core interactions between mitochondria and Aβ/tau, other molecular mechanisms (e.g., reduced mitochondrial biogenesis, calcium homeostasis imbalance) also contribute to mitochondrial dysfunction in AD, as discussed in section 3.3.

### 3.3 Mitochondrial biosynthesis reduction, calcium homeostasis imbalance, and other abnormal molecular mechanisms

#### 3.3.1 Reduced mitochondrial biosynthesis

From a mechanistic perspective, mitochondrial dysfunction in AD also involves reduced biosynthesis, calcium homeostasis imbalance, and dysregulation of molecular pathways. Although direct imaging evidence is lacking, potential imaging research directions can still be identified. Specifically, decreased mitochondrial biosynthesis is associated with downregulation of the peroxisome proliferator-activated receptor gamma coactivator-1 alpha (PGC-1α) signaling pathway, while reduced AMPK/Sirt1 activity weakens its activating effects. Currently, noninvasive assessment of these processes primarily relies on techniques such as ^31^P-MRS, but the clinical application faces significant challenges. Therefore, future development of PET tracers targeting PGC-1α or detection of ATP synthesis capacity via ^31^P-MRS may indirectly reflect biosynthetic levels (Skupienski et al., 2020).

First, from the perspective of current applications, although ^31^P-MRS is currently the only noninvasive and quantitative technique for evaluating high-energy phosphate (e.g., ATP) metabolism in the living brains, its clinical application remains far from widespread, primarily confined to the research field (Nam, 2025). A survey of neuroradiologists explicitly identified core barriers hindering their routine clinical application, including prolonged acquisition time, low spatial resolution, and the lack of standardized cross-center operational protocols (Nam, 2025). To address these limitations, future development directions are likely to focus on technological optimization and integration (Nam, 2025). For ^31^P-MRS, the key lies in developing faster imaging sequences and advanced reconstruction algorithms to reduce scan time and improve SNR, while promoting the establishment of standardized operational protocols to provide reliable dynamic biomarkers for evaluating the efficacy of mitochondrial-targeted therapies (Nam, 2025).

#### 3.3.2 Calcium homeostasis disruption

From the perspective of calcium homeostasis, Aβ induces Ca^2+^ influx through the MCU, leading to calcium overload and activation of persistent mPTP opening, ultimately resulting in membrane potential collapse and release of cytochrome c (Misrani et al., 2021). As a result, calcium dysregulation further promotes ROS generation, inhibits ATP synthesis, and activates the caspase cascade apoptotic pathway (Ebrahimi et al., 2025; Sun J. et al., 2025). Regarding calcium homeostasis imaging, it remains in the preclinical exploratory proof-of-concept stage, representing the future frontier for achieving *in vivo* specific monitoring of mitochondrial calcium dynamics (Li et al., 2020). Despite the dual challenges of probe development and imaging technology, its breakthrough could open a new window for understanding the core pathological mechanisms of AD (Moret et al., 2025). Current methods include MRS for detecting intracellular free calcium concentration (indirectly reflecting calcium overload) (Lutz and Bernard, 2018). The future high-field-strength MRI (≥7T) technology envisioned in the preceding text holds promise for achieving dynamic mitochondrial calcium imaging (Thiabaud et al., 2023).

#### 3.3.3 Metal ion homeostasis disorder

Imbalances in other metal ion dyshomeostasis constitute another important mechanism. For instance, copper ion (Cu^2+^) overload inhibits cAMP response element-binding protein (CREB) phosphorylation, reduces the expression of brain-derived neurotrophic factor (BDNF) and mitochondrial membrane potential; long-term exposure to iron ions (Fe) can directly induce mitochondrial damage and neuronal loss; zinc ions (Zn), on the other hand, participate in the pathological process by exacerbating mitochondrial damage and promoting p-tau liquid-liquid phase separation (Liu et al., 2023). The steady-state imbalance of the aforementioned metal ions can lead to mitochondrial damage.

In the assessment of metal ion homeostasis, the QSM mentioned earlier has advanced significantly and is now widely recognized as a critical tool for studying brain iron metabolism (Wang et al., 2025). To differentiate from section 3.1.2, this section focuses solely on the advantages, disadvantages, and application prospects of QSM technology in metal ion detection. For instance, in clinical studies of PD and AD, QSM has been employed to quantify iron deposition in specific brain regions (e.g., the putamen), with results showing a significant correlation with cognitive impairment (Wang et al., 2025). QSM has been used to quantify iron concentration, and future development of specific susceptibility imaging techniques for Cu^2+^ and Zn^2+^ may be feasible (Ding et al., 2025; Liu et al., 2018; Wu et al., 2023). However, QSM technology also has limitations, as its measurements are susceptible to variations in scanning equipment and reconstruction algorithms, leading to reproducibility issues. Additionally, it currently cannot distinguish between specific molecular forms of iron (e.g., ferritin and hemosiderin), which restricts the detailed interpretation of pathological mechanisms (Nam, 2025). To address these limitations, future development directions are clearly focused on technological optimization and integration (Nam, 2025). For QSM, future efforts will prioritize enhancing the reproducibility and reliability of measurements through optimized post-processing workflows. Studies have shown that in the complex image processing workflow of QSM, different algorithm choices significantly influence the sensitivity of results, making it crucial to establish consensus-based standard procedures (Salman et al., 2025). A longer-term perspective involves multimodal fusion, which entails spatially correlating the iron deposition information revealed by QSM with the Aβ and tau pathology demonstrated by PET and the energy metabolism status reflected by ^31^P-MRS, thereby constructing a more comprehensive pathological assessment model for AD (Wang et al., 2025).

#### 3.3.4 Other core molecular mechanisms: MAM dysfunction, SIRT pathway dysregulation, and impaired mitochondrial quality control

Mitochondrial dysfunction in AD also involves the dysregulation of multiple other core molecular mechanisms. First, abnormal endoplasmic reticulum (ER)-mitochondria interactions are primarily manifested as MAM dysfunction (Liu et al., 2023). MAM serves as a key regulatory hub, primarily responsible for regulating lipid synthesis, mitochondrial dynamics, ER stress, and calcium ion transmission. Among these, mutations in Mfn2 are directly associated with the occurrence of NDs, while Aβ promotes cell apoptosis by inhibiting the transfer of calcium ions from the ER to mitochondria, leading to elevated levels of ROS (Liu et al., 2023).

Second, dysregulation of the sirtuin (SIRT) signaling pathway is another important mechanism in AD. Specifically, SIRT1 alleviates Aβ-induced oxidative stress and promotes mitochondrial biogenesis by activating the AMPK/Nrf2 pathway, while also inhibiting the formation of Aβ oligomers and nuclear factor-kappa B (NF-κB) inflammatory signaling. SIRT3, on the other hand, improves mitochondrial oxygen consumption and ATP generation capacity by restoring nicotinamide adenine dinucleotide dehydrogenase subunit 2/nicotinamide adenine dinucleotide dehydrogenase subunit 4 (ND2/ND4) expression, and effectively mitigates Aβ-mediated mitochondrial dysfunction (Liu et al., 2023). At the imaging level, ultra-high field strength MRS ( ≥ 7T) can detect NAD(H) (a SIRT substrate), providing a metabolic indicator for SIRT pathway activity (Guo et al., 2024).

In addition, non-ubiquitin-dependent pathways also malfunction, with impaired mitochondrial protein receptor function leading to blocked clearance of damaged mitochondria, further exacerbating dysregulation of the mitochondrial quality control system. These molecular mechanisms are interconnected, collectively forming a complex network underlying mitochondrial dysfunction in AD, providing an important molecular basis for developing targeted therapeutic strategies.

**FIGURE 2:**
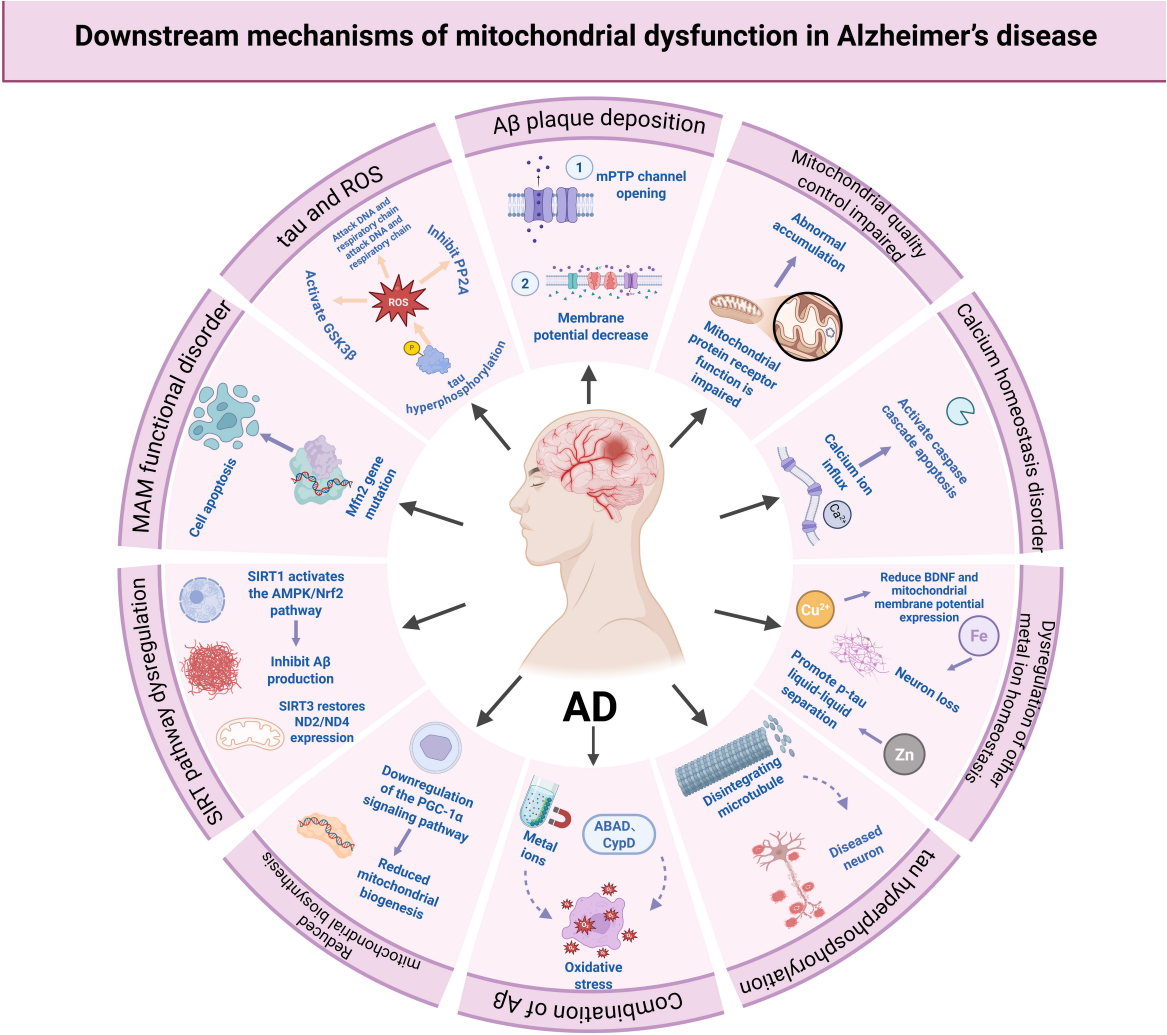
Downstream mechanisms of mitochondrial dysfunction in Alzheimer’s disease. Downstream mechanisms of Alzheimer’s disease (AD) include: mitochondrial dysfunction is presented as a bidirectional cascade driven by β-amyloid (Aβ) and mitochondria. In the Aβ-driven aspect, Aβ can directly impair the activity of respiratory chain enzymes or bind to mitochondrial proteins and cyclophilin D (CypD) to induce oxidative stress and apoptosis, as well as elevate cytoplasmic calcium levels, leading to calcium ion imbalance and mitochondrial dysfunction. On the other hand, mitochondrial dysfunction results in reduced cytochrome c oxidase (COX) activity in nonbrain tissues such as platelets and fibroblasts in AD patients. Additionally, mitochondrial DNA (mtDNA) can accelerate Aβ deposition. In terms of reduced mitochondrial biogenesis, decreased AMPK/Sirt1 activity and metabolic regulation imbalance (such as nutritional status, calcium ions, ROS, etc.) lead to weakened peroxisome proliferator-activated receptor gamma coactivator-1 alpha (PGC-1α) expression, thereby reducing Mfn2 expression and affecting mitochondrial renewal. Furthermore, in other core molecular mechanisms, there is an imbalance in endoplasmic reticulum (ER)-mitochondria interactions, such as dysfunction of mitochondria-associated endoplasmic reticulum membranes (MAMs) leading to insufficient regulation of lipid synthesis and ER stress, and abnormalities in key proteins like mitofusin-2 causing neurodegenerative diseases (NDs). Moreover, dysregulation of the Sirtuin (SIRT) pathway is also important, such as SIRT3 restoring ND2/ND4 expression and adenosine triphosphate (ATP) production. Additionally, imbalance in the homeostasis of metal ions like Cu^2+^, Fe, Zn further exacerbates mitochondrial damage through multiple pathways. (created with BioRender.com).

In summary, this section outlines other mitochondrial-related molecular dysregulation mechanisms in AD beyond energy and oxidative stress, such as reduced biosynthesis, calcium homeostasis imbalance, and SIRT pathway dysregulation and so on (Liu et al., 2023; Misrani et al., 2021). Currently, direct and specific *in vivo* imaging evidence for these specific mechanisms remains lacking, representing a significant research gap. Existing indirect or potential imaging modalities face challenges: ^31^P-MRS, while capable of indirectly reflecting ATP synthesis capacity to assess biosynthetic levels, is limited in clinical application due to prolonged acquisition time, low resolution, and lack of standardization (Jett et al., 2023); QSM can quantify brain iron concentration, which is associated with cognitive impairment, but its measurements are susceptible to equipment and algorithm biases, exhibit poor reproducibility, and cannot distinguish specific molecular forms of iron (Onukwufor et al., 2022). For calcium homeostasis, PGC-1α pathway activity, and NAD(H) levels, there are currently no mature and specific clinical imaging methods available. Future research directions are clear and challenging. First, it is essential to continue optimizing the scanning sequences and standardization protocols of existing technologies (e.g., ^31^P-MRS, QSM) to enhance their reliability and clinical applicability. Second, efforts should be dedicated to developing novel specific molecular probes, such as PET or MRI probes targeting mitochondrial calcium ions, PGC-1α, or specific metal ions (Cu^2+^, Zn). Third, the potential of ultra-high field strength ( ≥ 7T) MRS in detecting related metabolites (e.g., NAD(H), a substrate of the SIRT pathway) should be actively explored (Jett et al., 2023). The ultimate goal is to conduct multimodal correlation analysis between these imaging information reflecting different molecular dimensions and Aβ and tau pathological imaging, thereby comprehensively revealing the complex network of mitochondrial dysfunction in AD at the systemic level. Supplementary Table S2 summarizes the advantages and disadvantages of neuroimaging techniques related to mitochondrial dysfunction. Figure 2 describes the downstream mechanisms of mitochondrial dysfunction in AD.

## 4 Discussion

**FIGURE 3:**
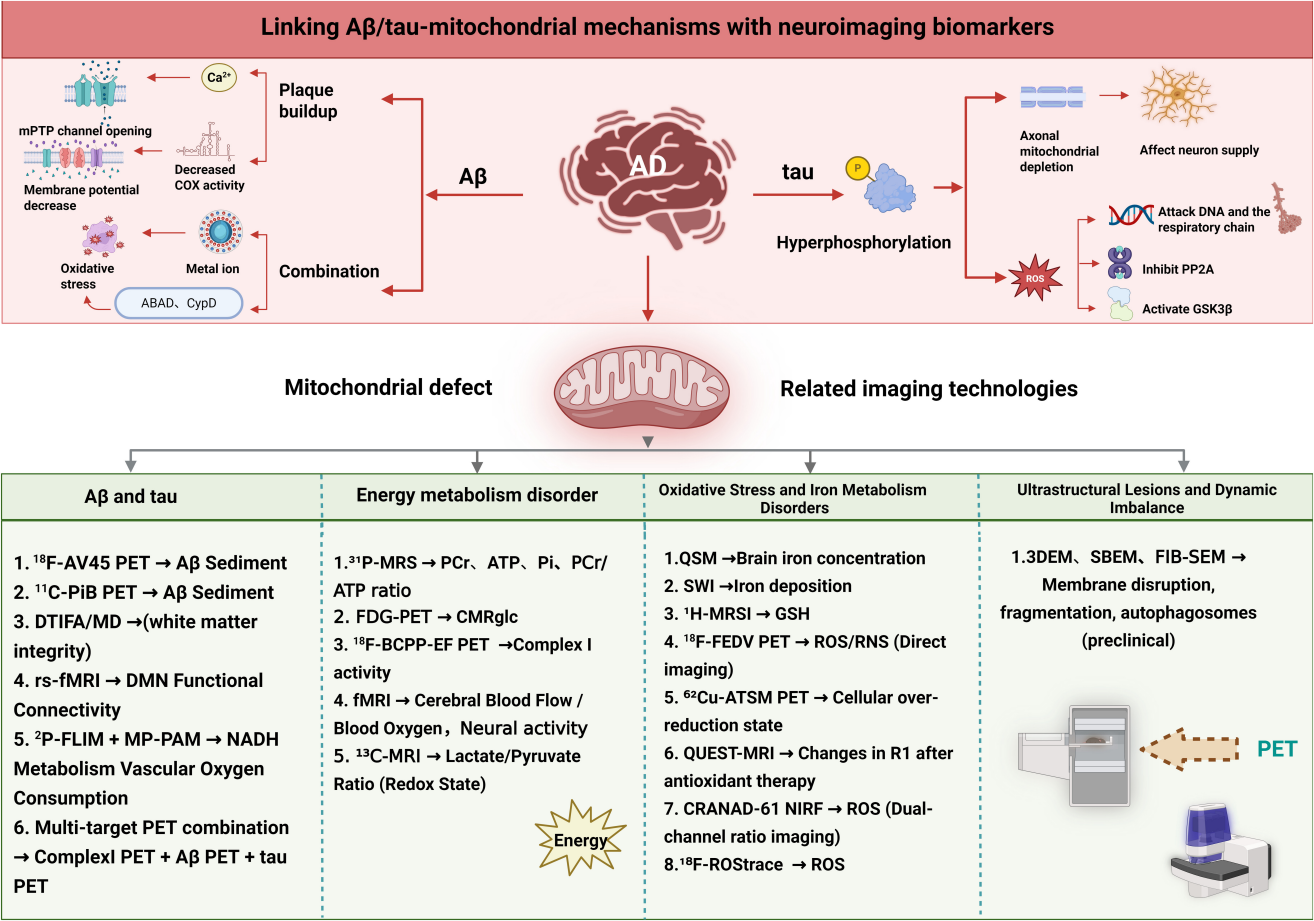
Integrating mitochondrial defects with Aβ/tau pathology and corresponding neuroimaging biomarkers. This figure integrates the pathological relationships between mitochondrial defects and β-amyloid (Aβ) and tau pathology in Alzheimer’s disease (AD) with their corresponding neuroimaging biomarkers. The left panel shows that Aβ plaque buildup induces mitochondrial dysfunction through mitochondrial permeability transition pore (mPTP) opening, decreased membrane potential, oxidative stress, metal ion interactions, Aβ-binding alcohol dehydrogenase (ABAD) and cyclophilin D (CypD) binding, and decreased cytochrome c oxidase (COX) activity. Tau hyperphosphorylation causes axonal mitochondrial depletion, attacks DNA/respiratory chain, inhibits protein phosphatase 2A (PP2A), and activates glycogen synthase kinase-3 beta (GSK3β). These converge on mitochondrial defects, forming a vicious cycle with Aβ and tau pathologies. The right panel organizes neuroimaging technologies into four categories: Aβ and tau pathology (^18^F-AV45 PET, ^11^C-PiB PET, DTI-FA/MD, rs-fMRI, ^2^P-FLIM+MP-PAM, multi-target PET), energy metabolism disorders (^31^P-MRS, FDG-PET, ^18^F-BCPP-EF PET, fMRI, ^13^C-MRI), oxidative stress and iron metabolism disorders (QSM, SWI, ^1^H-MRSI, ^18^F-FEDV PET, ^18^F-ROStrace, ^62^Cu-ATSM PET, QUEST-MRI, CRANAD-61, NIRF), and ultrastructural lesions (3DEM, SBEM, FIB-SEM). Together, these imaging modalities link mitochondrial defects with Aβ and tau pathology across molecular, metabolic, structural, and functional dimensions. ^18^F-AV45 PET: [^18^F]florbetaben positron emission tomography; ^11^C-acetate PET: carbon-11 acetate positron emission tomography imaging; DTI: diffusion tensor imaging; FA/MD: fractional anisotropy/mean diffusivity; rs-fMRI: resting-state functional magnetic resonance imaging; ^2^P-FLIM: two-photon fluorescence lifetime imaging; MP-PAM: multi-parameter photoacoustic microscopy imaging; PET: positron emission tomography; ^31^P-MRS: phosphorus-31 magnetic resonance spectroscopy; FDG-PET: fluorodeoxyglucose positron emission tomography; ^18^F-BCPP-EF: ^18^F-2-tert-butyl-4-chloro-5-{6-[2-(2-fluoroethoxy)-ethoxy]-pyridin-3-ylmethoxy-2H-pyridazin-3-one; fMRI: functional magnetic resonance imaging; ^13^C-MRI: hyperpolarized carbon-13 magnetic resonance imaging; QSM: quantitative susceptibility mapping; SWI: susceptibility-weighted imaging; ^1^H-MRSI: proton magnetic resonance spectroscopy imaging; ^18^F-FEDV: 2-deoxy-2-[^18^F]fluoro-α-D-arabinofuranosylvinyluracil; ^62^Cu-ATSM: copper-62 diacetyl-bis(N4-methylthiosemicarbazone); QUEST-MRI: QUEnching-assiSTed magnetic resonance imaging; CRANAD-61: 2-(4′-dimethylaminophenyl)-benzothiazolyl-derived cyanine dye; NIRF: near-infrared fluorescence imaging; 3DEM: three-dimensional electron microscopy; SBEM: serial block-face scanning electron microscopy; FIB-SEM: focused ion beam scanning electron microscope; ^11^C-PiB: carbon-11 pittsburgh compound-B. (created with BioRender.com).

This review systematically elucidates the multidimensional mechanisms of mitochondrial dysfunction in AD and its interactions with Aβ and tau pathology (Picard and Shirihai, 2022; Swerdlow, 2018). The upstream mechanisms are characterized by bioenergetic defects, oxidative stress, and mitochondrial dynamics imbalance, while downstream mechanisms involve bidirectional cascade reactions among Aβ, tau, and mitochondria (Swerdlow, 2018), along with reduced biosynthesis, calcium homeostasis disruption, and other abnormal molecular mechanisms (Liu et al., 2023; Misrani et al., 2021). Neuroimaging techniques provide crucial evidence for these mechanisms: for example, ^31^P-MRS and FDG-PET jointly reveal abnormal brain energy metabolism (Parasoglou et al., 2022); emerging QUEST-MRI and ^13^C-MRI technologies indirectly assess oxidative stress states (Berkowitz, 2018; Xiang et al., 2023); while DTI and rs-fMRI correlate mitochondrial dysfunction with damage to white matter networks and the DMN (Griffiths and Grant, 2023; Jett et al., 2023; Warren and Moustafa, 2023). Figure 3 describes the combination of mitochondrial defects with Aβ/tau pathological changes and the corresponding neuroimaging biomarkers.

However, current neuroimaging techniques still have significant limitations. Most methods are indirect d etection techniques, such as FDG-PET, which cannot evaluate the OXPHOS process (Jett et al., 2023), SWI has difficulty distinguishing Fe^2+^/Fe^3 +^ ions (Dietrich et al., 2017). Emerging direct probes (e.g., CRANAD-61,^18^F-FEDV) show promise, but their safety in humans and quantitative standardization require further validation (Xiang et al., 2023; Yang et al., 2017). Furthermore, cutting-edge technologies such as high-resolution 3DEM remain in the exploratory phase. Their clinical application faces challenges due to limitations in sample preparation, data analysis complexity, and equipment costs (Colasuonno et al., 2017).

Future research may focus on the following cutting-edge directions: First, developing highly specific probes to target mitochondrial complex activity, calcium dynamics, and specific ROS, thereby advancing their clinical translation. Second, establishing multimodal imaging fusion systems that integrate multidimensional information on energy metabolism, oxidative stress, protein pathology, and network function to build precise AD classification and prediction models. Third, utilizing noninvasive imaging as biomarkers to evaluate mitochondrial-targeted intervention strategies (e.g., enhancing mitophagy or regulating kinetic equilibrium) in clinical trials, thereby driving AD treatment toward personalized and precision medicine.

### 4.1 Key challenges and future directions

Despite the significant advancements in neuroimaging techniques that have profoundly deepened our understanding of mitochondrial dysfunction in AD, the field still faces a series of core challenges in methodology, data interpretation, and clinical translation. Systematically identifying and addressing these challenges is crucial for advancing mitochondria-targeted precision diagnosis and treatment of AD.

#### 4.1.1 Contradictions and heterogeneity in imaging findings

A prominent issue in current research is the frequent contradictions and heterogeneity in results across different imaging studies, which directly undermines the reliability and generalizability of relevant biomarkers. Specifically, this inconsistency manifests at multiple levels: At the molecular imaging level, the PET tracer ^18^F-BCPP-EF targeting mitochondrial complex I exhibits variable performance across different disease models. While demonstrating potential in AD research, its declining trend in PD models was not statistically significant, suggesting that dysfunction patterns may be disease-specific or that tracer sensitivity is influenced by pathological context (Mansur et al., 2020; Tsukada et al., 2014; Wilson et al., 2020). At the mechanistic research level, findings regarding the expression of PINK1/Parkin, the core pathway of mitophagy, in AD also vary across studies. This may be attributed to the brain region-specific distribution, low endogenous expression, and technical sensitivity of the PINK1 protein itself (Liu et al., 2025; Yoboue and Valente, 2020). Additionally, in metabolic imaging, although ^31^P-MRS is a key tool for evaluating brain energy metabolism, the lack of standardization in scanning protocols, ROI definitions, and quantification methods across studies significantly impairs the comparability and interpretative value of results (Jett et al., 2023; Rietzler et al., 2022). These contradictions and variations highlight the urgency of standardizing imaging acquisition and analysis workflows and validating them in clinically stratified cohorts.

#### 4.1.2 Methodological and translational bottlenecks in current neuroimaging technologies

Most existing neuroimaging techniques have inherent methodological limitations in revealing early and subtle mitochondrial pathology in AD, constituting a major bottleneck for clinical translation. The primary challenge lies in the indirect and nonspecific nature of many techniques. For example, the commonly used FDG-PET only reflects cerebral glucose uptake instead of the dominant OXPHOS process (Chia et al., 2025); SWI can detect iron deposition but cannot differentiate Fe^2+^ (involved in the Fenton reaction) from inert Fe^3 +^, which hinders the in-depth clarification of oxidative stress mechanisms (Dietrich et al., 2017). Second, the limitations in spatiotemporal resolution hinder the capture of early subcellular lesions. ^31^P-MRS is constrained by low spatial resolution and SNR, making it difficult to precisely quantify key small structures such as the hippocampus (Jett et al., 2023); while high-resolution EM techniques (e.g., 3DEM) that provide nanoscale structural information are currently limited to preclinical research due to the extreme complexity of sample preparation and data analysis (Colasuonno et al., 2017). Third, the availability and standardization of novel specific probes remain insufficient. Many promising direct detection probes face translational challenges: for instance, the PET ligand ^62^Cu-ATSM exhibits an extremely short half-life and poor accessibility due to oxidative stress (Ikawa et al., 2020); the automated synthesis yield and quantification standards of the novel probe ^18^F-FEDV require further optimization (Wilde et al., 2025); the NIRF probe CRANAD-61 is limited by tissue penetration depth (Ashleigh et al., 2023).

#### 4.1.3 Key research gaps and future priorities

To address these challenges, future research should focus on filling critical gaps and advancing translational progress in the field. First, longitudinal dynamic imaging studies are urgently needed. Current evidence predominantly comes from cross-sectional designs, with a severe lack of prospective studies that can track the full spectrum of AD and elucidate the dynamic interactions between oxidative stress, mitochondrial metabolism, and Aβ and tau pathology over time. This is crucial for clarifying causal timelines and identifying intervention windows (Green et al., 2024). Second, the integration of multimodal imaging and multidimensional biomarkers must be deepened. Combining multimodal data—such as ^31^P-MRS reflecting energy metabolism, Aβ and tau-PET for protein pathology, resting-state fMRI for network function, and DTI for white matter microstructure—with biomarkers in bodily fluids may enable the development of more precise disease classification and predictive models (Jett et al., 2023; Warren and Moustafa, 2023). Finally, sustained technological innovation and large-scale clinical validation are the cornerstones of translation. This includes developing probes with higher specificity (e.g., distinguishing iron valence states), leveraging AI to optimize the entire imaging workflow, and ultimately systematically validating the diagnostic efficacy, prognostic value, and therapeutic monitoring potential of new technologies in diverse large clinical cohorts to realize their true translational value.

### 4.2 Clinical translation barriers in mitochondrial imaging

Although the aforementioned neuroimaging techniques provide powerful tools for revealing mitochondrial pathology in AD, their translation from research to routine clinical application still faces a series of structural barriers.

Technological and methodological barriers remain the primary bottleneck. Many core technologies have not yet matured sufficiently to support large-scale clinical deployment. For instance, ^31^P-MRS, which enables noninvasive assessment of cerebral energy metabolism, is currently limited to research applications due to its low sensitivity, prolonged scanning time (typically exceeding 30 minutes), insufficient spatial resolution, and lack of standardized protocols across centers, making it difficult to accurately quantify key small brain regions such as the hippocampus (Jett et al., 2023). Similarly, QSM, while capable of evaluating iron deposition, is highly sensitive to magnetic field homogeneity and employs diverse post-processing algorithms, compromising the reproducibility and reliability of results (Nam, 2025). Emerging direct detection probes, such as the NIRF probe CRANAD-61, are constrained by tissue penetration depth, posing challenges for deep brain imaging in humans (Ashleigh et al., 2023).

The accessibility and standardization of tracers severely limit the application of molecular imaging. Promising oxidative stress PET tracers, such as ^62^Cu-ATSM, must rely on on-site radionuclide generators due to their extremely short half-life (approximately 9.7 min), resulting in poor accessibility and hindering widespread clinical implementation (Ikawa et al., 2020). Novel probes like ^18^F-FEDV, despite demonstrating excellent performance in preclinical models, suffer from low automated synthesis yields and lack unified imaging and quantification standards, remaining in preclinical or early clinical research stages, far from routine clinical application (Wilde et al., 2025). Additionally, insufficient standardization of scanning protocols and data analysis workflows across studies leads to significant heterogeneity in results, impeding the establishment of consensus on biomarkers (Jett et al., 2023).

Furthermore, the challenges of cost and resource allocation cannot be overlooked (Kanabur et al., 2026; Li et al., 2026). The production and quality control of high-field magnetic resonance (e.g., 7T), PET/MRI multimodal devices, and novel radiotracer agents entail substantial equipment investments and high per-visit costs. These factors limit the widespread adoption of such technologies in resource-constrained settings, potentially exacerbating disparities in healthcare accessibility (Fabius et al., 2025; Li et al., 2026).

Finally, the time-consuming regulatory and validation processes are also one of the reasons for difficulties in clinical translation. Any novel imaging biomarker or probe must undergo rigorous multicenter, large-sample clinical trials to demonstrate diagnostic efficacy, reproducibility, and clinical utility before being approved for clinical diagnosis or efficacy evaluation. This process is time-consuming and labor-intensive, representing a critical hurdle that must be overcome for ultimate translation (Hu et al., 2025; Parums, 2025).

In conclusion, advancing the clinical translation of mitochondrial imaging requires not only continuous technological innovation to overcome methodological limitations, but also interdisciplinary collaboration focused on tracer development, standardization of imaging protocols, cost control, and rigorous clinical validation. These efforts are essential to transform these promising tools into clinically valuable instruments for early AD diagnosis and precision medicine.
