# Supplementary material for: Mitochondrial dysfunction in Alzheimer’s disease: connecting pathophysiology with neuroimaging
Source: Front Aging Neurosci. 2026 May 29;18:1748227. doi: 10.3389/fnagi.2026.1748227 (PMC13260646; doi:10.3389/fnagi.2026.1748227)
Supplement: Supplementary file 1 [file Data_Sheet_1.docx]

Supplementary Materials

**Table S1: Markers of mitochondrial signaling.**

| **Type of markers** | **Names of markers** | **Some important signaling** | **Key functions** | **References** |
| --- | --- | --- | --- | --- |
| Energetics | ATP/ADP | AMPK signaling;  Purinergic receptors | Energy sensing;  Inflammatory regulation | (Di Virgilio et al., 2018; Sies and Jones 2020) |
|  | Pi | Energy sensing;  Indicator of energy demand stress | Reflects cellular energy status;  product of ATP hydrolysis | (Jett et al., 2023; Onukwufor et al., 2022; Swerdlow 2018) |
|  | PCr | Energy sensing;  ATP regeneration | Immediate energy reserve;  Rapid ATP regeneration | (Jett et al., 2023; Onukwufor et al., 2022; Swerdlow 2018) |
|  | NAD+ | Sirtuin; PARP | Metabolic regulation;  DNA repair | (Bai et al., 2011; Imai and Guarente 2014) |
|  | NADP+ / NADPH | Glutathione system (GSH/GSSG);  Lipid synthesis | Antioxidant defense;  Reductive power supply | (Kračun et al., 2025; Lu and Holmgren 2014) |
|  | FAD+ | ETC Complex II;  Oxidoreductases | Electron transport;  Redox balance | (Forman and Zhang 2021; Sun et al., 2005) |
| Metabolites | Succinate | HIF-1α pathway;  SUCNR1 inflammatory pathway | Hypoxia adaptation;  Inflammatory regulation | (Mills et al., 2016) |
|  | α-Ketoglutarate | Epigenetic modification;  mTORC1 regulation | Gene expression regulation;  Metabolic adaptation | (Carey et al., 2015) |
|  | Acetyl-CoA | Histone acetylation;  mTORC1 activation | Epigenetics;  Cell growth | (Lee et al., 2018) |
| Ions | Ca²⁺ | MCU-mediated metabolic activation; mPTP apoptosis | Energy metabolism;  Cell death regulation | (Baughman et al., 2011; Giorgio et al., 2018) |
|  | H⁺ | Proton gradient-driven ATP synthesis | Core mechanism of oxidative phosphorylation | (Kühlbrandt 2015) |
|  | K⁺ | Mitochondrial KATP channels and ischemic preconditioning | ROS regulation;  Cell protection | (Szabo and Szewczyk 2023) |
|  | Na⁺ | NCLX-mediated Ca²⁺ efflux | Calcium homeostasis | (Palty et al., 2010) |
|  | Mg²⁺ | Enzyme cofactors (α-KGDH, ATP synthase) | Regulation of metabolic enzyme activity | (Romani 2011) |
|  | Cu2+ / Cu+ | Copper overloading system for complex IV subunits;  Lipoylation of TCA protein | Cuproptosis | (Tsvetkov et al., 2022) |
|  | Fe²⁺/Fe³⁺ | Fenton reaction | Promotes excessive mitochondrial fission | (Onukwufor et al., 2022) |
| Heat | / | / | Thermogenesis;  Metabolic adaptation;  Stress protection;  Protein homeostasis | (El-Gammal et al., 2022; Nedergaard and Cannon 2014; Vihervaara et al., 2018) |
| Steroid hormones | ERβ | ERβ-NRF2/PGC-1α pathway | Antioxidant defense;  Mitochondrial biogenesis | (Capllonch-Amer et al., 2014; Liao et al., 2015; Ventura-Clapier et al., 2017) |
|  | GR | GR-ETC complex transcription;  GR-Bax/Bak-mPTP cell death regulation;  ETC complexes I/III and SOD2/GPx;  PGC-1α inhibition and Drp1 phosphorylation;  NCLX-mediated Ca²⁺ efflux | Oxidative phosphorylation;  Apoptosis regulation;  Mitochondrial quantity and quality regulation;  Oxidative stress and defence;  Calcium homeostasis | (Besseiche et al., 2015; Cain and Cidlowski 2017; Du et al., 2009; Kokkinopoulou and Moutsatsou 2021; Schmidt et al., 2004) |
|  | AR | AR-mTOR-OXPHOS pathway | Muscle metabolism enhancement;  Mitochondrial energy optimization | (Basualto-Alarcón et al., 2013; White et al., 2013) |
| Mitochondrial- Derived Peptides (MDPs) | Humanin | AKT/STAT3; AMPK/mTOR;  JAK/STAT;  Bax signaling;  GH/IGF-1 axis | Apoptosis regulation;  Metabolic control;  ER stress, Neuroprotection;  Aging regulation | (Hazafa et al., 2021; Merry et al., 2020; Muzumdar et al., 2009; Sreekumar et al., 2017; Thiankhaw et al., 2022) |
|  | MOTS-c | AMPK/Nrf2 | Insulin sensitization;  Antioxidant defense | (Lee et al., 2015; Muzumdar et al., 2009; Zheng et al., 2023) |
|  | SHLPs | CXCR7/ MAPK/ERK | Oxidative stress;  Energy homeostasis | (Kim et al., 2023) |
| Vesicles | Mitochondrial-derived vesicles (MDVs) | Screening of damaged components;  Activation of autophagy | Maintaining mitochondrial integrity;  Quality control and autophagy | (White et al., 2013) |
|  |  | Transporting mtDNA/DAMP | Immune activation;  Inflammation regulation | (Nakahira et al., 2011; West et al., 2015) |
|  |  | Mediating mitochondria-lysosome/ER communication | Lipid metabolism;  Calcium signaling coordination | (Basso et al., 2018; Wong et al., 2018) |
| DNA | mtDNA | cGAS-STING;  NLRP3 inflammasome | Immune activation;  Inflammation regulation | (Sansone et al., 2017) |
| ROS and gases | H₂O₂ | NF-κB pathway;  HIF-1α pathway | Inflammation regulation;  Hypoxia adaptation | (Chandel et al., 1998; Morgan and Liu 2011) |
|  | O₂⁻ | PINK1/Parkin autophagy;  SOD antioxidant | Mitochondrial quality control;  Oxidative stress feedback | (Narendra et al., 2010) |
|  | NO | COX inhibition;  ONOO-generation | Respiratory regulation;  Protein nitrification damage | (Moncada and Erusalimsky 2002; Radi 2004) |
|  | ROS | Nrf2/ARE;  mPTP apoptosis / cytochrome c / caspase cascade reaction;  HIF-1α pathway;  NF-κB pathway;  PTEN pathway;  PINK1/Parkin autophagy | Oxidative Stress Response.  Proliferation and Apoptosis;  Inflammation regulation;  Mitochondrial quality control | (Ma 2013; Tait and Green 2010) |

AMP-activated protein kinase; PARP: Poly (ADP-ribose) polymerase; GSH/GSSG: Glutathione (reduced)/Glutathione disulfide (oxidized); ETC: Electron transport chain; HIF-1α: Hypoxia-inducible factor 1-alpha; SUCNR1: Succinate Receptor 1; mTORC1: Mammalian target of rapamycin complex 1; MCU: Mitochondrial calcium uniporter; mPTP: Mitochondrial Permeability Transition Pore; KATP: ATP-sensitive potassium channel; NCLX: Sodium/calcium/lithium exchanger; α-KGDH: Alpha-ketoglutarate dehydrogenase; Erβ: Estrogen Receptor beta; NRF2: Nuclear factor erythroid 2-related factor 2; PGC-1α: Peroxisome proliferator-activated receptor gamma coactivator 1-alpha; GR: Glucocorticoid Receptor; Bax/Bak: BCL2-associated X protein / BCL2-antagonist/killer 1; Drp1: Dynamin-related protein 1; AKT/STAT3: Protein Kinase B / Signal Transducer and Activator of Transcription 3; JAK/STAT: Janus kinase / Signal Transducer and Activator of Transcription; GH/IGF-1: Growth hormone / insulin-like growth factor 1; mtDNA: Mitochondrial DNA; cGAS-STING: Cyclic GMP-AMP synthase / Stimulator of interferon genes; NF-κB: Nuclear Factor kappa-light-chain-enhancer of activated B cells; SOD: Superoxide dismutase; COX: Cytochrome c oxidase; ONOO⁻: peroxynitrite; ARE: Antioxidant response element; PTEN: Phosphatase and tensin homolog; TCA: Tricarboxylic Acid Cycle; Pcr: Creatine phosphate; Pi: Inorganic Phosphate

**Table S2.** **Imaging-related techniques**

| **Technical category** | **Technical name** | **English abbreviation** | **Advantages and disadvantages** | **References** |
| --- | --- | --- | --- | --- |
| energy metabolism assessment | Phosphorus-31 Magnetic Resonance Spectroscopy | ³¹P-MRS | Advantages: The only non-invasive method to detect OXPHOS metabolites; Disadvantages: Low signal-to-noise ratio, limited spatial resolution. | (Qiao et al., 2006) |
|  | Functional Magnetic Resonance Imaging | fMRI | Advantages: High spatial resolution, can reflect brain activity; Disadvantages: Indirect indicator, cannot directly measure metabolites. | (Butterfield and Halliwell 2019) |
|  | Magnetic transfer phosphorescence magnetic resonance spectroscopic imaging | ³¹P-MT-MRSI | Advantages: Can assess the regulatory effect of drugs on energy metabolism; Disadvantages: Technically complex, limited clinical application. | (Prasuhn et al., 2022) |
|  | Mitochondrial complex I-targeted PET imaging | ¹⁸F-BCPP-EF PET | Advantages: High specificity, good reproducibility; Disadvantages: Only show trends, not very significant. | (Tsukada) |
|  | Broadband near-infrared spectroscopy | bNIRS | Advantages: portable, can be used at the bedside; disadvantages: low spatial resolution, easily interfered with. | (Lange et al., 2019) |
|  | Hyperpolarized Carbon-13 Magnetic Resonance Imaging | ¹³C-MRI | Advantages: Can directly detect metabolic products; Disadvantages: High equipment requirements, complex preparation of tracers. | (Sharma et al., 2024) |
|  | Acetate PET imaging | ^11^C-acetate PET | Advantages: Can assess oxidative metabolism; Disadvantages: Limited application in the nervous system, mainly used for tumors. | (Song et al., ) |
|  | Fluorodeoxyglucose positron emission tomography | FDG-PET | Advantages: Can detect brain glucose metabolic rate early. Disadvantages: Unable to assess the OXPHOS process, which accounts for 90% of brain ATP production. | (de Leon et al., 1983; Foster et al., 1983; Friedland et al., 1983; Haidar et al., 2023; Vaishnavi et al., 2010) |
| Oxidative stress assessment | Proton magnetic resonance spectroscopy imaging | ¹H-MRSI | Advantages: non-invasive, can dynamically monitor treatment effects; Disadvantages: spectral peaks tend to overlap, requires special editing techniques. | (Mandal et al., 2015; Mandal et al., 2019; Mischley et al., 2016) |
|  | Oxidative stress PET imaging | ⁶²Cu-ATSM PET | Advantages: Can be used for in vivo assessment of oxidative stress; Disadvantages: Short tracer half-life, high equipment requirements | (Donnelly et al., 2012; Ikawa et al., 2020; Yoshii et al., 2012) |
|  | Magnetic susceptibility-weighted imaging | SWI | Advantages: sensitive to iron deposition; Disadvantages: unable to distinguish between Fe²⁺ and Fe³⁺ | (Biasiotto et al., 2019) (Dietrich et al., 2017) |
|  | Quenching-assisted MRI | QUEST-MRI | Advantages: No need for contrast agents, high spatial resolution; Disadvantages: The technology is relatively new, insufficient clinical validation. | (Berkowitz 2018) |
|  | Oxidative stress PET imaging | ¹⁸F-FEDV PET | Advantages: Possesses broad-spectrum reactive oxygen species (RONS) reactivity, excellent blood-brain barrier (BBB) penetration, and high stability; Disadvantages: Requires radioactive tracers. | (Wilde et al., 2025) |
|  | ¹⁸F-ROStrace | ¹⁸F-ROStrace | Advantages: The first PET tracer for direct quantitative and dynamic visualization of ROS in the brain; exhibits high selectivity for superoxide anions and low background uptake in normal tissues; shows a high degree of consistency with signal levels of oxidative stress-related biological pathological indicators;  Disadvantages: Unable to distinguish between mitochondrial and cytoplasmic ROS sources; easily influenced by blood perfusion and systemic inflammatory status, and lacking arterial blood sample correction. | (Gallagher et al., 2025; Park et al., 2026) |
|  | Quantitative susceptibility mapping | QSM | Advantages: Quantitative iron concentration related to cognitive decline; No radioactive tracer is required and there is no radiation risk; Comprehensive assessment of oxygen metabolism and blood flow status in brain tissue; Disadvantages: High requirement for magnetic field uniformity | (Onukwufor et al., 2022) |
|  | Near-infrared fluorescence imaging | NIRF | Advantages: Can distinguish active lesions, high sensitivity; Disadvantages: Requires injection of fluorescent probes, higher invasiveness. | (Yang et al., 2017) |
|  | CRANAD-61 | CRANAD-61 | Advantages:multilayer imaging; rapid response to reactive oxygen species; good blood-brain barrier penetration and physiologicalstability; Disadvantages:Human body safety and quantitative standardization still need to be verified | (Yang et al., 2017) |
|  | Two-photon fluorescence lifetime imaging | ^2^P-FLIM | Advantages: Early changes can be observed in live animals; Disadvantages: Equipment is expensive, and it is more invasive. | (Norambuena et al., 2024) |
|  | Multi-parameter photoacoustic microscopy imaging | MP-PAM | Advantages: Can simultaneously evaluate vascular and metabolic conditions; Disadvantages: Limited spatial penetration depth. | (Norambuena et al., 2024) |
| Structural assessment | Diffusion tensor imaging | DTI | Advantages: Can assess fiber bundle integrity; Disadvantages: Sensitive to artifacts, limited resolution. | (Griffiths and Grant 2023; Jett et al., 2023) |
|  | Resting-state functional magnetic resonance imaging | rs-fMRI | Advantages: Can reveal neural network anomalies; Disadvantages: Indirect indicator, influenced by multiple factors. | (Warren and Moustafa 2023) |
|  | beta-amyloid PET imaging | ¹⁸F-AV45 PET | Advantages: High specificity, can diagnose early; Disadvantages:unable to assess mitochondrial function. | (Barthel 2025.6.1; Clark et al., 2011) |
|  | High-resolution electron microscopy technology | 3DEM | Advantages: Can reconstruct mitochondrial cristae membrane structure. | (Ju et al., 2023) |
|  | Serial block-face scanning electron microscopy | SBEM | Advantages: Suitable for high-throughput analysis of large-volume samples (such as axon bundles);  Disadvantages:Low resolution | (Choi et al., 2020) |
|  | Focused ion beam scanning electron microscope | FIB-SEM | Advantages: Combines high resolution with large volume analysis capability. | (Choi et al., 2020) |

ATP: Adenosine Triphosphate; OXPHOS: oxidative phosphorylation; RONS: Reactive Oxygen and Nitrogen Species; BBB: blood-brain barrier; ³¹P-MRS: phosphorus-31 magnetic resonance spectroscopy; fMRI: functional magnetic resonance imaging; ³¹P-MT-MRSI: phosphorus-31 magnetization transfer magnetic resonance spectroscopic imaging; ^18^F-BCPP-EF: 18F-2-tert-butyl-4-chloro-5-{6-[2-(2-fluoroethoxy)-ethoxy]-pyridin-3-ylmethoxy-2H-pyridazin-3-one; ¹³C-MRI: hyperpolarized carbon-13 magnetic resonance imaging; ^11^C-acetate PET: carbon-11 acetate positron emission tomography imaging; FDG-PET: fluorodeoxyglucose positron emission tomography; ¹H-MRSI: proton magnetic resonance spectroscopy imaging; ^62^Cu-ATSM: copper-62 diacetyl-bis(N4-methylthiosemicarbazone); SWI: susceptibility-weighted imaging; QUEST-MRI: QUEnching-assiSTed magnetic resonance imaging; ^18^F-FEDV: 2-deoxy-2-[¹⁸F]fluoro-α-D-arabinofuranosylvinyluracil; QSM: quantitative susceptibility mapping; NIRF: near-infrared fluorescence imaging; ^2^P-FLIM: two-photon fluorescence lifetime imaging; MP-PAM: multi-parameter photoacoustic microscopy imaging; DTI: diffusion tensor imaging; rs-fMRI: resting-state functional magnetic resonance imaging; ¹⁸F-AV45 PET: [¹⁸F]florbetaben positron emission tomography; 3DEM: three-dimensional electron microscopy; SBEM: serial block-face scanning electron microscopy; FIB-SEM: focused ion beam scanning electron microscope; PCr: phosphocreatine; Pi: inorganic phosphate; CCO: cytochrome c oxidase; GSH: glutathione; ROS: reactive oxygen species; Aβ: β-amyloid; AD: Alzheimer’s disease; CMRO2: cortical microvascular oxygenation; FA/MD: fractional anisotropy/mean diffusivity; OEF: oxygen uptake fraction; EM: electron microscopy; qBOLD: quantitative blood oxygenation level dependent; CRANAD-61: 2-(4'-dimethylaminophenyl)-benzothiazolyl-derived cyanine dye; ETC: electron transport chain; CBF: cerebral blood flow; PET: positron emission tomography.

**References:**

Bai, P., Cantó, C., Oudart, H., Brunyánszki, A., Cen, Y., Thomas, C., et al. (2011). PARP-1 inhibition increases mitochondrial metabolism through SIRT1 activation. Cell Metab. 13, 461-468. doi: 10.1016/j.cmet.2011.03.004

Barthel, H., et al. (2025.6.1). Phase III study of 18F-florbetaben amyloid PET imaging in Alzheimer's disease and other dementias. doi:

Basso, V., Marchesan, E., Peggion, C., Chakraborty, J., von Stockum, S., Giacomello, M., et al. (2018). Regulation of ER-mitochondria contacts by Parkin via Mfn2. Pharmacol Res. 138, 43-56. doi: 10.1016/j.phrs.2018.09.006

Basualto-Alarcón, C., Jorquera, G., Altamirano, F., Jaimovich, E. and Estrada, M. (2013). Testosterone signals through mTOR and androgen receptor to induce muscle hypertrophy. Med Sci Sports Exerc. 45, 1712-1720. doi: 10.1249/MSS.0b013e31828cf5f3

Baughman, J. M., Perocchi, F., Girgis, H. S., Plovanich, M., Belcher-Timme, C. A., Sancak, Y., et al. (2011). Integrative genomics identifies MCU as an essential component of the mitochondrial calcium uniporter. Nature. 476, 341-345. doi: 10.1038/nature10234

Berkowitz, B. A. (2018). Oxidative stress measured in vivo without an exogenous contrast agent using QUEST MRI. J Magn Reson. 291, 94-100. doi: 10.1016/j.jmr.2018.01.013

Besseiche, A., Riveline, J. P., Gautier, J. F., Bréant, B. and Blondeau, B. (2015). Metabolic roles of PGC-1α and its implications for type 2 diabetes. Diabetes & Metabolism. 41, 347-357. doi: <https://doi.org/10.1016/j.diabet.2015.02.002>

Biasiotto, G., Filosto, M. and Zanella, I. (2019). Editorial: Iron and Neurodegeneration. Front Neurosci. 13, 1382. doi: 10.3389/fnins.2019.01382

Butterfield, D. A. and Halliwell, B. (2019). Oxidative stress, dysfunctional glucose metabolism and Alzheimer disease. Nat Rev Neurosci. 20, 148-160. doi: 10.1038/s41583-019-0132-6

Cain, D. W. and Cidlowski, J. A. (2017). Immune regulation by glucocorticoids. Nat Rev Immunol. 17, 233-247. doi: 10.1038/nri.2017.1

Capllonch-Amer, G., Sbert-Roig, M., Galmés-Pascual, B. M., Proenza, A. M., Lladó, I., Gianotti, M., et al. (2014). Estradiol stimulates mitochondrial biogenesis and adiponectin expression in skeletal muscle. J Endocrinol. 221, 391-403. doi: 10.1530/joe-14-0008

Carey, B. W., Finley, L. W., Cross, J. R., Allis, C. D. and Thompson, C. B. (2015). Intracellular α-ketoglutarate maintains the pluripotency of embryonic stem cells. Nature. 518, 413-416. doi: 10.1038/nature13981

Chandel, N. S., Maltepe, E., Goldwasser, E., Mathieu, C. E., Simon, M. C. and Schumacker, P. T. (1998). Mitochondrial reactive oxygen species trigger hypoxia-induced transcription. Proc Natl Acad Sci U S A. 95, 11715-11720. doi: 10.1073/pnas.95.20.11715

Choi, S. H., Kim, K. Y., Perkins, G. A., Phan, S., Edwards, G., Xia, Y., et al. (2020). AIBP protects retinal ganglion cells against neuroinflammation and mitochondrial dysfunction in glaucomatous neurodegeneration. Redox Biol. 37, 101703. doi: 10.1016/j.redox.2020.101703

Clark, C. M., Schneider, J. A., Bedell, B. J., Beach, T. G., Bilker, W. B., Mintun, M. A., et al. (2011). Use of florbetapir-PET for imaging beta-amyloid pathology. Jama. 305, 275-283. doi: 10.1001/jama.2010.2008

de Leon, M. J., Ferris, S. H., George, A. E., Christman, D. R., Fowler, J. S., Gentes, C., et al. (1983). Positron emission tomographic studies of aging and Alzheimer disease. AJNR Am J Neuroradiol. 4, 568-571. doi:

Di Virgilio, F., Sarti, A. C., Falzoni, S., De Marchi, E. and Adinolfi, E. (2018). Extracellular ATP and P2 purinergic signalling in the tumour microenvironment. Nat Rev Cancer. 18, 601-618. doi: 10.1038/s41568-018-0037-0

Dietrich, O., Levin, J., Ahmadi, S.-A., Plate, A., Reiser, M. F., Bötzel, K., et al. (2017). MR imaging differentiation of Fe2+ and Fe3+ based on relaxation and magnetic susceptibility properties. Neuroradiology. 59, 403-409. doi: 10.1007/s00234-017-1813-3

Donnelly, P. S., Liddell, J. R., Lim, S., Paterson, B. M., Cater, M. A., Savva, M. S., et al. (2012). An impaired mitochondrial electron transport chain increases retention of the hypoxia imaging agent diacetylbis(4-methylthiosemicarbazonato)copperII. Proc Natl Acad Sci U S A. 109, 47-52. doi: 10.1073/pnas.1116227108

Du, J., Wang, Y., Hunter, R., Wei, Y., Blumenthal, R., Falke, C., et al. (2009). Dynamic regulation of mitochondrial function by glucocorticoids. Proc Natl Acad Sci U S A. 106, 3543-3548. doi: 10.1073/pnas.0812671106

El-Gammal, Z., Nasr, M. A., Elmehrath, A. O., Salah, R. A., Saad, S. M. and El-Badri, N. (2022). Regulation of mitochondrial temperature in health and disease. Pflügers Archiv - European Journal of Physiology. 474, 1043-1051. doi: 10.1007/s00424-022-02719-2

Forman, H. J. and Zhang, H. (2021). Targeting oxidative stress in disease: promise and limitations of antioxidant therapy. Nat Rev Drug Discov. 20, 689-709. doi: 10.1038/s41573-021-00233-1

Foster, N. L., Chase, T. N., Fedio, P., Patronas, N. J., Brooks, R. A. and Di Chiro, G. (1983). Alzheimer's disease: focal cortical changes shown by positron emission tomography. Neurology. 33, 961-965. doi: 10.1212/wnl.33.8.961

Friedland, R. P., Budinger, T. F., Ganz, E., Yano, Y., Mathis, C. A., Koss, B., et al. (1983). Regional cerebral metabolic alterations in dementia of the Alzheimer type: positron emission tomography with [18F]fluorodeoxyglucose. J Comput Assist Tomogr. 7, 590-598. doi: 10.1097/00004728-198308000-00003

Gallagher, E., Li, S., Lee, H., Xu, H., Lee, V. M., Mach, R. H., et al. (2025). Noninvasive Detection of Oxidative Stress in a Mouse Model of 4R Tauopathy via Positron Emission Tomography with [(18)F]ROStrace. Int J Mol Sci. 26, doi: 10.3390/ijms26051845

Giorgio, V., Guo, L., Bassot, C., Petronilli, V. and Bernardi, P. (2018). Calcium and regulation of the mitochondrial permeability transition. Cell Calcium. 70, 56-63. doi: <https://doi.org/10.1016/j.ceca.2017.05.004>

Griffiths, J. and Grant, S. G. N. (2023). Synapse pathology in Alzheimer's disease. Semin Cell Dev Biol. 139, 13-23. doi: 10.1016/j.semcdb.2022.05.028

Haidar, H., Majzoub, R. E., Hajeer, S. and Abbas, L. A. (2023). Arterial spin labeling (ASL-MRI) versus fluorodeoxyglucose-PET (FDG-PET) in diagnosing dementia: a systematic review and meta-analysis. BMC Neurol. 23, 385. doi: 10.1186/s12883-023-03432-y

Hazafa, A., Batool, A., Ahmad, S., Amjad, M., Chaudhry, S. N., Asad, J., et al. (2021). Humanin: A mitochondrial-derived peptide in the treatment of apoptosis-related diseases. Life Sci. 264, 118679. doi: 10.1016/j.lfs.2020.118679

Ikawa, M., Okazawa, H., Nakamoto, Y. and Yoneda, M. (2020). PET Imaging for Oxidative Stress in Neurodegenerative Disorders Associated with Mitochondrial Dysfunction. Antioxidants (Basel). 9, doi: 10.3390/antiox9090861

Imai, S. and Guarente, L. (2014). NAD+ and sirtuins in aging and disease. Trends Cell Biol. 24, 464-471. doi: 10.1016/j.tcb.2014.04.002

Jett, S., Boneu, C., Zarate, C., Carlton, C., Kodancha, V., Nerattini, M., et al. (2023). Systematic review of (31)P-magnetic resonance spectroscopy studies of brain high energy phosphates and membrane phospholipids in aging and Alzheimer's disease. Front Aging Neurosci. 15, 1183228. doi: 10.3389/fnagi.2023.1183228

Ju, W. K., Perkins, G. A., Kim, K. Y., Bastola, T., Choi, W. Y. and Choi, S. H. (2023). Glaucomatous optic neuropathy: Mitochondrial dynamics, dysfunction and protection in retinal ganglion cells. Prog Retin Eye Res. 95, 101136. doi: 10.1016/j.preteyeres.2022.101136

Kim, S. K., Tran, L. T., NamKoong, C., Choi, H. J., Chun, H. J., Lee, Y.-h., et al. (2023). Mitochondria-derived peptide SHLP2 regulates energy homeostasis through the activation of hypothalamic neurons. Nature Communications. 14, 4321. doi: 10.1038/s41467-023-40082-7

Kokkinopoulou, I. and Moutsatsou, P. (2021). Mitochondrial Glucocorticoid Receptors and Their Actions. Int J Mol Sci. 22, doi: 10.3390/ijms22116054

Kračun, D., Lopes, L. R., Cifuentes-Pagano, E. and Pagano, P. J. (2025). NADPH oxidases: redox regulation of cell homeostasis and disease. Physiol Rev. 105, 1291-1428. doi: 10.1152/physrev.00034.2023

Kühlbrandt, W. (2015). Structure and function of mitochondrial membrane protein complexes. BMC Biology. 13, 89. doi: 10.1186/s12915-015-0201-x

Lange, F., Dunne, L., Hale, L. and Tachtsidis, I. (2019). MAESTROS: A Multiwavelength Time-Domain NIRS System to Monitor Changes in Oxygenation and Oxidation State of Cytochrome-C-Oxidase. IEEE J Sel Top Quantum Electron. 25, 7100312. doi: 10.1109/jstqe.2018.2833205

Lee, C., Zeng, J., Drew, B. G., Sallam, T., Martin-Montalvo, A., Wan, J., et al. (2015). The mitochondrial-derived peptide MOTS-c promotes metabolic homeostasis and reduces obesity and insulin resistance. Cell Metab. 21, 443-454. doi: 10.1016/j.cmet.2015.02.009

Lee, J. V., Berry, C. T., Kim, K., Sen, P., Kim, T., Carrer, A., et al. (2018). Acetyl-CoA promotes glioblastoma cell adhesion and migration through Ca(2+)-NFAT signaling. Genes Dev. 32, 497-511. doi: 10.1101/gad.311027.117

Liao, T. L., Tzeng, C. R., Yu, C. L., Wang, Y. P. and Kao, S. H. (2015). Estrogen receptor-β in mitochondria: implications for mitochondrial bioenergetics and tumorigenesis. Ann N Y Acad Sci. 1350, 52-60. doi: 10.1111/nyas.12872

Lu, J. and Holmgren, A. (2014). The thioredoxin antioxidant system. Free Radic Biol Med. 66, 75-87. doi: 10.1016/j.freeradbiomed.2013.07.036

Ma, Q. (2013). Role of nrf2 in oxidative stress and toxicity. Annu Rev Pharmacol Toxicol. 53, 401-426. doi: 10.1146/annurev-pharmtox-011112-140320

Mandal, P. K., Saharan, S., Tripathi, M. and Murari, G. (2015). Brain glutathione levels--a novel biomarker for mild cognitive impairment and Alzheimer's disease. Biol Psychiatry. 78, 702-710. doi: 10.1016/j.biopsych.2015.04.005

Mandal, P. K., Shukla, D., Tripathi, M. and Ersland, L. (2019). Cognitive Improvement with Glutathione Supplement in Alzheimer's Disease: A Way Forward. J Alzheimers Dis. 68, 531-535. doi: 10.3233/jad-181054

Merry, T. L., Chan, A., Woodhead, J. S. T., Reynolds, J. C., Kumagai, H., Kim, S. J., et al. (2020). Mitochondrial-derived peptides in energy metabolism. Am J Physiol Endocrinol Metab. 319, E659-e666. doi: 10.1152/ajpendo.00249.2020

Mills, E. L., Kelly, B., Logan, A., Costa, A. S. H., Varma, M., Bryant, C. E., et al. (2016). Succinate Dehydrogenase Supports Metabolic Repurposing of Mitochondria to Drive Inflammatory Macrophages. Cell. 167, 457-470.e413. doi: 10.1016/j.cell.2016.08.064

Mischley, L. K., Conley, K. E., Shankland, E. G., Kavanagh, T. J., Rosenfeld, M. E., Duda, J. E., et al. (2016). Central nervous system uptake of intranasal glutathione in Parkinson's disease. NPJ Parkinsons Dis. 2, 16002. doi: 10.1038/npjparkd.2016.2

Moncada, S. and Erusalimsky, J. D. (2002). Does nitric oxide modulate mitochondrial energy generation and apoptosis? Nature Reviews Molecular Cell Biology. 3, 214-220. doi: 10.1038/nrm762

Morgan, M. J. and Liu, Z. G. (2011). Crosstalk of reactive oxygen species and NF-κB signaling. Cell Res. 21, 103-115. doi: 10.1038/cr.2010.178

Muzumdar, R. H., Huffman, D. M., Atzmon, G., Buettner, C., Cobb, L. J., Fishman, S., et al. (2009). Humanin: a novel central regulator of peripheral insulin action. PLoS One. 4, e6334. doi: 10.1371/journal.pone.0006334

Nakahira, K., Haspel, J. A., Rathinam, V. A., Lee, S. J., Dolinay, T., Lam, H. C., et al. (2011). Autophagy proteins regulate innate immune responses by inhibiting the release of mitochondrial DNA mediated by the NALP3 inflammasome. Nat Immunol. 12, 222-230. doi: 10.1038/ni.1980

Narendra, D. P., Jin, S. M., Tanaka, A., Suen, D. F., Gautier, C. A., Shen, J., et al. (2010). PINK1 is selectively stabilized on impaired mitochondria to activate Parkin. PLoS Biol. 8, e1000298. doi: 10.1371/journal.pbio.1000298

Nedergaard, J. and Cannon, B. (2014). The browning of white adipose tissue: some burning issues. Cell Metab. 20, 396-407. doi: 10.1016/j.cmet.2014.07.005

Norambuena, A., Sagar, V. K., Wang, Z., Raut, P., Feng, Z., Wallrabe, H., et al. (2024). Disrupted mitochondrial response to nutrients is a presymptomatic event in the cortex of the APP<sup>SAA</sup> knock-in mouse model of Alzheimer’s disease. bioRxiv. 2024.2002.2002.578668. doi: 10.1101/2024.02.02.578668

Onukwufor, J. O., Dirksen, R. T. and Wojtovich, A. P. (2022). Iron Dysregulation in Mitochondrial Dysfunction and Alzheimer's Disease. Antioxidants (Basel). 11, doi: 10.3390/antiox11040692

Palty, R., Silverman, W. F., Hershfinkel, M., Caporale, T., Sensi, S. L., Parnis, J., et al. (2010). NCLX is an essential component of mitochondrial Na+/Ca2+ exchange. Proc Natl Acad Sci U S A. 107, 436-441. doi: 10.1073/pnas.0908099107

Park, J. Y., Park, S. M., Lee, T. S., Lee, S. J., Kim, J. Y., Oh, S. J., et al. (2026). Noninvasive PET imaging of LPS-induced oxidative stress in skeletal muscle using a ROS-targeting radiotracer. Sci Rep. 16, 4917. doi: 10.1038/s41598-026-35489-3

Qiao, H., Zhang, X., Zhu, X. H., Du, F. and Chen, W. (2006). In vivo 31P MRS of human brain at high/ultrahigh fields: a quantitative comparison of NMR detection sensitivity and spectral resolution between 4 T and 7 T. Magn Reson Imaging. 24, 1281-1286. doi: 10.1016/j.mri.2006.08.002

Radi, R. (2004). Nitric oxide, oxidants, and protein tyrosine nitration. Proc Natl Acad Sci U S A. 101, 4003-4008. doi: 10.1073/pnas.0307446101

Romani, A. M. (2011). Cellular magnesium homeostasis. Arch Biochem Biophys. 512, 1-23. doi: 10.1016/j.abb.2011.05.010

Sansone, P., Savini, C., Kurelac, I., Chang, Q., Amato, L. B., Strillacci, A., et al. (2017). Packaging and transfer of mitochondrial DNA via exosomes regulate escape from dormancy in hormonal therapy-resistant breast cancer. Proc Natl Acad Sci U S A. 114, E9066-e9075. doi: 10.1073/pnas.1704862114

Schmidt, S., Rainer, J., Ploner, C., Presul, E., Riml, S. and Kofler, R. (2004). Glucocorticoid-induced apoptosis and glucocorticoid resistance: molecular mechanisms and clinical relevance. Cell Death Differ. 11 Suppl 1, S45-55. doi: 10.1038/sj.cdd.4401456

Sharma, G., Duarte, S., Shen, Q. and Khemtong, C. (2024). Analyses of mitochondrial metabolism in diseases: a review on (13)C magnetic resonance tracers. RSC Adv. 14, 37871-37885. doi: 10.1039/d4ra03605k

Sies, H. and Jones, D. P. (2020). Reactive oxygen species (ROS) as pleiotropic physiological signalling agents. Nature Reviews Molecular Cell Biology. 21, 363-383. doi: 10.1038/s41580-020-0230-3

Song, Z., Xie, X., Chen, Y., Zhang, B., Li, X., Yang, Y., et al. Innate immune sensing of Z-nucleic acids by ZBP1-RIPK1 axis drives neuroinflammation in Alzheimer&#x2019;s disease. Immunity. doi: 10.1016/j.immuni.2025.07.024

Sreekumar, P. G., Hinton, D. R. and Kannan, R. (2017). Endoplasmic reticulum-mitochondrial crosstalk: a novel role for the mitochondrial peptide humanin. Neural Regen Res. 12, 35-38. doi: 10.4103/1673-5374.198970

Sun, F., Huo, X., Zhai, Y., Wang, A., Xu, J., Su, D., et al. (2005). Crystal structure of mitochondrial respiratory membrane protein complex II. Cell. 121, 1043-1057. doi: 10.1016/j.cell.2005.05.025

Swerdlow, R. H. (2018). Mitochondria and Mitochondrial Cascades in Alzheimer's Disease. J Alzheimers Dis. 62, 1403-1416. doi: 10.3233/jad-170585

Szabo, I. and Szewczyk, A. (2023). Mitochondrial Ion Channels. Annu Rev Biophys. 52, 229-254. doi: 10.1146/annurev-biophys-092622-094853

Tait, S. W. and Green, D. R. (2010). Mitochondria and cell death: outer membrane permeabilization and beyond. Nat Rev Mol Cell Biol. 11, 621-632. doi: 10.1038/nrm2952

Thiankhaw, K., Chattipakorn, K., Chattipakorn, S. C. and Chattipakorn, N. (2022). Roles of humanin and derivatives on the pathology of neurodegenerative diseases and cognition. Biochim Biophys Acta Gen Subj. 1866, 130097. doi: 10.1016/j.bbagen.2022.130097

Tsukada, H., Ohba, H., Nishiyama, S., Kaneko, K., & Kakuchi, T. Novel 18F-Labeled Mitochondrial Complex I Inhibitor as a Potential Pet Agent for Brain Imaging. doi: 10.2967/jnumed.114.138842

Tsvetkov, P., Coy, S., Petrova, B., Dreishpoon, M., Verma, A., Abdusamad, M., et al. (2022). Copper induces cell death by targeting lipoylated TCA cycle proteins. Science. 375, 1254-1261. doi: 10.1126/science.abf0529

Vaishnavi, S. N., Vlassenko, A. G., Rundle, M. M., Snyder, A. Z., Mintun, M. A. and Raichle, M. E. (2010). Regional aerobic glycolysis in the human brain. Proc Natl Acad Sci U S A. 107, 17757-17762. doi: 10.1073/pnas.1010459107

Ventura-Clapier, R., Moulin, M., Piquereau, J., Lemaire, C., Mericskay, M., Veksler, V., et al. (2017). Mitochondria: a central target for sex differences in pathologies. Clin Sci (Lond). 131, 803-822. doi: 10.1042/cs20160485

Vihervaara, A., Duarte, F. M. and Lis, J. T. (2018). Molecular mechanisms driving transcriptional stress responses. Nature Reviews Genetics. 19, 385-397. doi: 10.1038/s41576-018-0001-6

Warren, S. L. and Moustafa, A. A. (2023). Functional magnetic resonance imaging, deep learning, and Alzheimer's disease: A systematic review. J Neuroimaging. 33, 5-18. doi: 10.1111/jon.13063

West, A. P., Khoury-Hanold, W., Staron, M., Tal, M. C., Pineda, C. M., Lang, S. M., et al. (2015). Mitochondrial DNA stress primes the antiviral innate immune response. Nature. 520, 553-557. doi: 10.1038/nature14156

White, J. P., Gao, S., Puppa, M. J., Sato, S., Welle, S. L. and Carson, J. A. (2013). Testosterone regulation of Akt/mTORC1/FoxO3a signaling in skeletal muscle. Molecular and Cellular Endocrinology. 365, 174-186. doi: <https://doi.org/10.1016/j.mce.2012.10.019>

Wilde, J. H., Sun, Y.-Y., Simpson, S. R., Hill, E. R., Fu, Z., Bian, E. J., et al. (2025). A positron emission tomography tracer for the imaging of oxidative stress in the central nervous system. Nature Biomedical Engineering. 9, 716-729. doi: 10.1038/s41551-025-01362-3

Wong, Y. C., Ysselstein, D. and Krainc, D. (2018). Mitochondria–lysosome contacts regulate mitochondrial fission via RAB7 GTP hydrolysis. Nature. 554, 382-386. doi: 10.1038/nature25486

Yang, J., Zhang, X., Yuan, P., Yang, J., Xu, Y., Grutzendler, J., et al. (2017). Oxalate-curcumin–based probe for micro- and macroimaging of reactive oxygen species in Alzheimer’s disease. Proceedings of the National Academy of Sciences. 114, 12384-12389. doi: doi:10.1073/pnas.1706248114

Yoshii, Y., Yoneda, M., Ikawa, M., Furukawa, T., Kiyono, Y., Mori, T., et al. (2012). Radiolabeled Cu-ATSM as a novel indicator of overreduced intracellular state due to mitochondrial dysfunction: studies with mitochondrial DNA-less ρ0 cells and cybrids carrying MELAS mitochondrial DNA mutation. Nucl Med Biol. 39, 177-185. doi: 10.1016/j.nucmedbio.2011.08.008

Zheng, Y., Wei, Z. and Wang, T. (2023). MOTS-c: A promising mitochondrial-derived peptide for therapeutic exploitation. Front Endocrinol (Lausanne). 14, 1120533. doi: 10.3389/fendo.2023.1120533
